# Community notes increase trust in fact-checking on social media

**DOI:** 10.1093/pnasnexus/pgae217

**Published:** 2024-05-31

**Authors:** Chiara Patricia Drolsbach, Kirill Solovev, Nicolas Pröllochs

**Affiliations:** JLU Giessen, 35394 Giessen, Germany; JLU Giessen, 35394 Giessen, Germany; JLU Giessen, 35394 Giessen, Germany

**Keywords:** misinformation, fact-checking, trust, social media

## Abstract

Community-based fact-checking is a promising approach to fact-check social media content at scale. However, an understanding of whether users trust community fact-checks is missing. Here, we presented n=1,810 Americans with 36 misleading and nonmisleading social media posts and assessed their trust in different types of fact-checking interventions. Participants were randomly assigned to treatments where misleading content was either accompanied by simple (i.e. context-free) misinformation flags in different formats (expert flags or community flags), or by textual “community notes” explaining why the fact-checked post was misleading. Across both sides of the political spectrum, community notes were perceived as significantly more trustworthy than simple misinformation flags. Our results further suggest that the higher trustworthiness primarily stemmed from the context provided in community notes (i.e. fact-checking explanations) rather than generally higher trust towards community fact-checkers. Community notes also improved the identification of misleading posts. In sum, our work implies that context matters in fact-checking and that community notes might be an effective approach to mitigate trust issues with simple misinformation flags.

**Significance Statement**

Social media providers have been called upon to develop effective countermeasures to combat the spread of misinformation on their platforms. However, a large proportion of users distrust professional fact-checkers and the stance on fact-checking is increasingly becoming a partisan issue. In this study, we demonstrate that community-based fact-checking systems (e.g. X’s Community Notes) that focus on providing fact-checking context have the potential to mitigate trust issues that are common in traditional approaches to fact-checking on social media. Fostering trust in fact-checking is vitally important, especially as we face emerging challenges due to AI-generated misinformation.

## Main

Concerns about misinformation on social media have been rising in recent years, particularly given its potential impact on elections (1–6), public health (7–10), and public safety (11–13). Major social media providers such as X (formerly Twitter) and Facebook have thus been called upon to develop effective countermeasures to combat the spread of misinformation on their platforms (14–18). To this end, a widely implemented approach is the use of professional fact-checkers to identify and label misleading posts (19, 20). The rationale is that if users are warned that a message is false, they should be less likely to believe it. Previous work has evaluated the effects of flags and warning labels on misinformation discernment and sharing intentions. Although some contradictory evidence exists (21), there is a growing consensus that it can be effective to put misinformation flags on content contested by fact-checkers (22–32); for a review, see Ref. (32).

However, current approaches to fact-checking social media content have several drawbacks that limit their full potential. First, due to the limited amount of fact-checks that experts can perform, they are unable to accommodate the amount and speed of content creation on social media (33, 34). A large proportion of misinformation on social media thus goes unchecked. Second, a large proportion of Americans have concerns regarding the independence of the experts’ assessment (35, 36), and the stance on fact-checking is increasingly becoming a partisan issue. Adherents of the left tend to be less tolerable of the spread of misinformation and have greater trust in fact-checking (37–40). According to surveys, a majority of Republican partisans (70%) and half of all U.S. adults believe that fact-checkers are biased and that their corrections cannot be trusted (36). While research indicates that fact-checks are broadly effective (31, 39, 41), their influence on users is smaller—although still significant—for those who distrust fact-checkers (39). Hence, even when a social media post gets fact-checked and flagged, the impact on users may be limited due to a lack of trust (42).

Given these problems regarding scalability and trust, recent works have proposed outsourcing fact-checking of social media content to the actual social media users, i.e. nonexpert fact-checkers in the crowd (33, 43–48); for a review, see Ref. (34). The rationale is that the “wisdom of crowds” (i.e. the aggregated assessments of nonexpert fact-checkers) could result in an accuracy that is comparable to that of experts (34, 49, 50). A prominent implementation of community-based fact-checking is X’s “Community Notes” feature (formerly known as “Birdwatch”) (51–55). This feature allows regular social media users on X to identify posts (formerly “tweets”) they believe are misleading and write (textual) annotations that provide *context* to the post, so-called “community notes” (throughout this article, we use the term “community notes” platform-agnostic to refer to textual fact-checking annotations carried out by community fact-checkers). Applying such a crowd-based approach to fact-checking social media posts has the potential to address the scalability issues with expert fact-checking. Compared to expert-based assessments, significantly larger quantities (51, 56) and a wider range of posts (57, 58) could be fact-checked at a higher speed (57). However, evidence on whether exposing users to community notes mitigates trust issues with simple misinformation flags is missing.

Two distinctive factors may lead one to expect that community notes may be perceived as more trustworthy than simple misinformation flags. First, users may be more willing to trust their peers (i.e. community fact-checkers) than experts. Americans’ trust in experts and established information sources has been in continued decline over the last years (59, 60). At the same time, people trust information sources more if they perceive the source as similar to themselves (61–64). Hence, higher (perceived) source credibility may render community fact-checks to be perceived as more trustworthy. Second, community notes allow fact-checkers to add context by explaining why the fact-checked post was misleading and linking to relevant external sources (51, 55). People’s trust in information increases with the amount of supporting evidence (61, 65, 66) and presenting users with additional information that directly refutes a false statement may help to debunk misinformation (67). While context-free misinformation flags may create a gap in a user’s mental state (e.g. “why is this post labeled as misleading?”), context refuting misleading claims may also fill in plausible details, which may strengthen the recall of correct information. Overall, both source effects (i.e. higher trust in peers) and the provision of additional context may make community notes to be perceived as more trustworthy than simple misinformation flags.

Previous work on community-based fact-checking has primarily focused on the accuracy of community fact-checkers. Experimental studies found that, while the assessment of individuals might be noisy and ineffective (50), the crowd can be quite accurate in identifying misleading social media content. The assessment of even relatively small crowds is comparable to those of experts (33, 34, 44, 45, 47, 48, 68). Despite challenges with politically motivated flagging (51, 52), research further shows that users, to a large extent, perceive community-created fact-checks for social media posts as being informative and helpful (51). Also, community fact-checkers surpass experts in the volume, variety, and speed of fact-checking (51, 56, 58, 69). Overall, prior studies suggest that community-based fact-checking may address the scalability problem of expert-based approaches. Yet, an understanding of whether users perceive community fact-checks as more trustworthy than misinformation flags is missing. Here, we add by assessing users’ trust in community fact-checks for social media posts.

In this study, we conducted a pre-registered survey experiment with n=1,810 American residents (politically balanced) to assess their trust in different types of fact-checking interventions (see Materials and methods section). Participants in our experiment were randomly assigned to treatments where misleading content was accompanied by context-free misinformation flags in different formats (expert flags or community flags), or by textual community notes explaining why the fact-checked post was misleading. Participants rated the perceived trustworthiness of each fact-check. We then implemented hierarchical regression models to analyze how trust in fact-checks varies across the different fact-checking interventions. Our objective is to understand whether people are more likely to trust community notes than simple misinformation flags and how the effects vary depending on the political concordance between participants and posts. We also examine source effects, i.e. whether fact-checks carried out by experts or the community are perceived as more trustworthy. This allows us to analyze whether trust in fact-checks is fostered by source effects (higher trust in peers) or the provision of additional context (i.e. fact-checking explanations). Furthermore, we study how users’ ability to identify misinformation varies across the different fact-checking interventions.

## Results

**Fig. 1.**
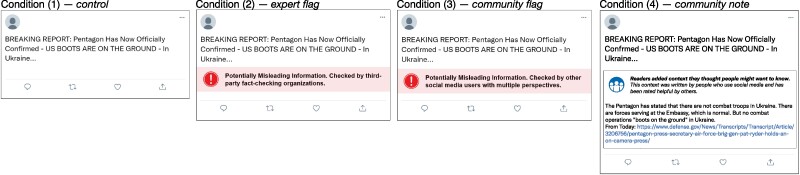
Example of a social media post and fact-checks shown to participants. Participants were randomly assigned to one of four conditions: (1) *Control*, in which the participants were presented only with the social media posts (i.e. without fact-checks), (2) *expert flag*, where misleading posts were supplemented by a flag indicating that expert fact-checkers categorized the content as being misleading, (3) *community flag*, where misleading posts were supplemented by a flag indicating that community fact-checkers categorized the content as being misleading, or (4) *community note*, where misleading posts were supplemented with textual community notes explaining why the information in the post is misleading. Posts were politically balanced to appeal to subjects with different political views (pro-Republican, pro-Democrat, and politically neutral).

Here, we investigate users’ trust in fact-checks for social media posts with a pre-registered (https://aspredicted.org/rb45k.pdf) four-condition survey experiment (see Materials and methods section). We presented n=1,810 American residents ($M_{\mathrm{age}}=42$ years, 51% female; politically balanced; see Supplementary Material A for further descriptive statistics) with 36 (18 misleading and 18 nonmisleading) social media posts and assessed their trust in different types of fact-checks. Participants were recruited via Prolific across multiple sessions and randomly assigned to a control, treatments where all misleading content was accompanied by simple (i.e. context-free) misinformation flags (expert flags and community flags), or a treatment where all misleading content was supplemented with textual community notes explaining why a post was misleading. Each community note in our experiment represented a real-world fact-check from X’s Community Notes platform that has been rated as helpful by multiple users with diverse viewpoints (see Materials and methods section). All posts and fact-checks were presented in a standard “X format” (see example in Fig. 1), and were prevalidated (see Supplementary Material E for details) to appeal to subjects with different political views (pro-Republican, pro-Democrat, and politically neutral). Following our preregistration, we further asked the participants for an assessment of the misleadingness of the posts. This allowed us to analyze how users’ ability to identify misinformation varied across the four conditions.

### Trust in fact-checks

**Fig. 2.**
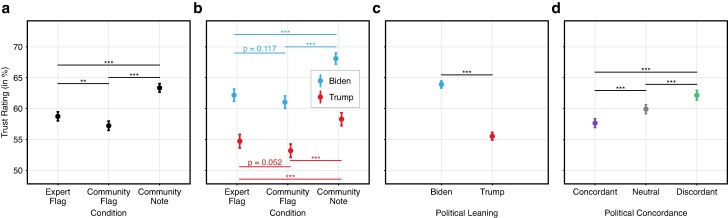
Trust in fact-checks for misleading posts. a) Mean trust ratings across fact-checking interventions, i.e. experimental conditions. Note that trust in fact-checks was only assessed if a fact-check was actually presented to users, i.e. across experimental conditions 2–4. b) Mean trust ratings across experimental conditions separated by participants’ political leanings (leaning Trump vs. Biden). c) Trust in fact-checks for participants leaning towards Trump vs. Biden. d) Trust in fact-checks for politically concordant, neutral, and discordant misleading posts. The 7-point Likert scale responses were normalized to the interval [0, 1]. The *y*-axis begins at 50%; the scale is the same across each panel. N= 24,003 observations across 1,347 participants. Statistical significance (^***^p<0.001; ^**^p<0.01; ^*^p<0.05) is calculated using two-tailed *t*-tests.

The data suggest significant differences in the perceived trustworthiness of the fact-checks presented to participants across the experimental conditions and political leanings. The participants’ mean trust ratings (7-point Likert scale normalized to the interval [0, 1]) are shown in Fig. 2. On average, simple expert flags were rated as slightly more trustworthy (M=0.59) than simple community flags (M=0.57). According to two-tailed *t*-tests, this difference was significant for the full sample (P=0.005), but failed to reached statistical significance when looking at Biden supporters (P=0.117) or Trump supporters (P=0.052) separately. Compared to expert flags, exposing participants to textual community notes that elaborate on the reasons on why the fact-checked post was misleading significantly increased (P<0.001) the perceived trustworthiness (M=0.63). This improvement was significantly higher (P<0.001) for Biden supporters (M=0.68) than for Trump supporters (M=0.58). We further observe that the average trust ratings differed according to the political orientation of both the participants and the fact-checked posts. Despite concerns with trust in fact-checkers (36), Trump-supporting participants rated the majority of fact-checks as at least somewhat trustworthy (M=0.56). However, participants preferring Biden exhibited a significantly higher (P<0.001) baseline trust in fact-checks (M=0.64). Furthermore, fact-checks were, on average, perceived as significantly (each P<0.001) less trustworthy if the misleading post was politically concordant (M=0.58) as opposed to misleading posts that were politically neutral (M=0.60) or politically discordant (M=0.62).

**Fig. 3.**
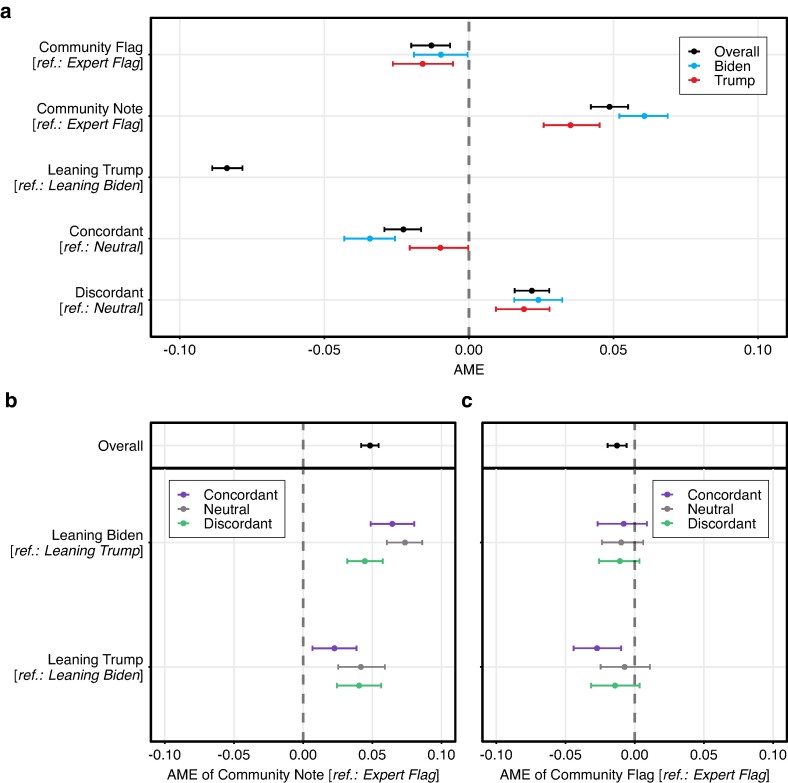
Community notes were rated as more trustworthy than simple misinformation flags. a) Shown are the average marginal effects (AME) and 95% confidence intervals from a hierarchical linear regression model with interaction terms predicting trust in fact-checks (7-point Likert scale normalized to the interval [0, 1]). AME is the difference in the average predicted trust ratings between the group of interest and the reference group (e.g. an AME of +0.05 indicates a 5 percentage point difference in trust ratings). b) AME of replacing an expert flag with a community note (i.e. the average difference between the predicted marginal effects for community notes vs. expert flags) across the political leanings of participants (leaning Trump vs. Biden) and the political congruence of the fact-checked posts (concordant, neutral, discordant). c) AME of replacing an expert flag with a community flag (i.e. the average difference between the predicted marginal effects for community flags vs. expert flags) across the political leanings of participants and the fact-checked posts. Control variables and random intercepts for posts and subjects were included. The 95% confidence intervals (error bars) were derived using the bootstrap method for 1,000 resamples. N= 24,003 observations across 1,347 participants. Full estimation results are in Tables S5 and S7.

Next, we fitted a hierarchical linear regression model with three-way interaction terms and random intercepts for subjects and posts to predict trustworthiness ratings (7-point Likert scale normalized to the interval [0, 1]). This allowed us to control for between-subject variations and study interaction effects (see Materials and methods section). Figure 3 visualizes the average marginal effects (AME) for our main explanatory variables (full estimation results are in Supplementary Material B. Consistent with the analysis of group-level means, the regression model predicts that community notes were perceived as significantly more trustworthy than simple expert flags. On average, replacing an expert flag with a community note would have increased trust in the fact-check by 4.8 percentage points (AME =0.048; 95% CI=[0.042,0.055]; P<0.001). In terms of percentages, this corresponds to an increase in trust of 8.2%. Moreover, simple community flags were slightly less trustworthy than simple expert flags (AME =−0.013; 95% CI=[−0.020,−0.006]; P=0.003). This suggests that the higher trustworthiness of community notes primarily stemmed from the additional context (i.e. the fact-checking explanations) rather than the fact-checking source.

The increases in trust varied across the political leanings of participants and posts (see Fig. 3b). For Biden supporters, replacing an expert flag with a community note would have increased trust in the fact-check by 7.4 percentage points (AME =0.074; 95% CI=[0.061,0.086]; P<0.001) for politically neutral posts, by 4.5 percentage points (AME =0.045; 95% CI=[0.032,0.058]; P<0.001) for politically discordant posts, and by 6.4 percentage points (AME =0.064; 95% CI=[0.049,0.080]; P<0.001) for politically concordant posts. For Trump supporters, replacing an expert flag with a community note would have increased trust in the fact-check by 4.2 percentage points (AME =0.042; 95% CI=[0.025,0.059]; P<0.001) for politically neutral posts, by 4.0 percentage points (AME =0.040; 95% CI=[0.024,0.056]; P<0.001) for politically discordant posts, and by 2.3 percentage points (AME =0.023; 95% CI=[0.007,0.039]; P=0.010) for politically concordant posts. Complementing earlier research on misinformation flagging (70), this implies that participants leaning towards Trump were more hesitant to trust the context provided in community notes when exposed to misinformation that was politically concordant (e.g. COVID-19 misinformation).

### Identification of misleading and nonmisleading posts

**Fig. 4.**
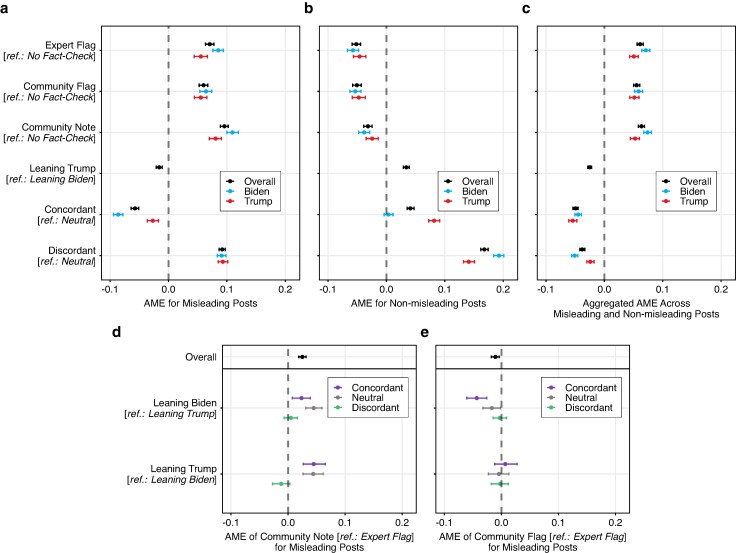
All fact-checking interventions improved the identification of misleading posts. Shown are the average marginal effects (AME) and 95% confidence intervals from a hierarchical linear regression model with interaction terms predicting the perceived misleadingness of a post (7-point Likert scale normalized to the interval [0, 1]). AME is the difference in the average predicted misleadingness ratings between the group of interest and the reference group (e.g. an AME of +0.05 indicates a 5 percentage point difference in perceived misleadingness). a) AMEs for misleading posts. b) AMEs for nonmisleading posts. c) Aggregated AMEs across misleading and nonmisleading posts (i.e. overall discernment). d) AMEs when replacing an expert flag with a community note (i.e. the average difference between the predicted marginal effects for community notes vs. expert flags) across the political leanings of participants (leaning Trump vs. Biden) and the political congruence of the fact-checked posts (concordant, neutral, discordant). e) AMEs when replacing an expert flag with a community flag (i.e. the average difference between the predicted marginal effects for community flags vs. expert flags) across the political leanings of participants and the fact-checked posts. Control variables and random intercepts for posts and subjects were included. The 95% confidence intervals (error bars) were derived using the bootstrap method for 1,000 resamples. N= 64,454 observations across 1,810 participants. Full estimation results are in Tables S6 and S8.

Next, we analyzed participants’ identification of misleading and nonmisleading posts. The majority of participants assigned to the control condition (in which no fact-check was shown) successfully identified misleading posts. The participants’ misleadingness ratings (7-point Likert scale normalized to the interval [0, 1]) for posts in the control condition were significantly higher (*t*-test: t=103.23; df=15207; P<0.001, two-tailed) if they were misleading (M=0.73) than if they were nonmisleading (M=0.44). Notably, for both misleading and nonmisleading posts, the share of posts rated as at least somewhat misleading was relatively high. A plausible explanation is that we asked participants for an assessment of misleadingness (i.e. a broad concept) rather than just true and false veracity (see Ref. (71)). To quantify the effects of the different fact-checking interventions (i.e. conditions 2–4) on the identification of misleading and nonmisleading posts, we fitted a hierarchical linear regression model with four-way interaction terms and random intercepts for subjects and posts to predict misleadingness ratings (see Materials and methods section). Figure 4 visualizes the average marginal effects (see Supplementary Material B for full estimation results).

All fact-checking interventions significantly improved the identification of misleading social media posts (see Fig. 4a,d,e). Compared to the control condition, displaying fact-checks increased the perceived misleadingness by, on average, 7.1 percentage points for expert flags (AME =0.071; 95% CI=[0.063,0.078]; P<0.001), 6.0 percentage points for community flags (AME =0.060; 95% CI=[0.053,0.067]; P<0.001), and 9.6 percentage points for community notes (AME =0.096; 95% CI=[0.089,0.102]; P<0.001). In terms of percentages, these numbers translate to an increase in perceived misleadingness of 9.7% for expert flags, 8.2% for community flags, and 13.1% for community notes. Hence, participants were more likely to correctly identify misleading posts if exposed to a community note vs. an expert flag (AME =0.025; 95% CI=[0.019,0.032]; P<0.001), and less likely if exposed to a community flag vs. an expert flag (AME =−0.011; 95% CI=[−0.018,−0004]; P=0.003). The efficacy of the fact-checking interventions was slightly but significantly (each P<0.01) larger for Biden supporters than for Trump supporters in the case of expert flags (Biden supporters: AME =0.085; 95% CI=[0.076,0.094]; P<0.001, Trump supporters: AME =0.055; 95% CI=[0.044,0.067]; P<0.001) and community notes (Biden supporters: AME =0.110; 95% CI=[0.100,0.120]; P<0.001, Trump supporters: AME =0.081; 95% CI=[0.070,0.091]; P<0.001), but did not significantly (P=0.443) differ across political leanings for community flags (Biden supporters: AME =0.064; 95% CI=[0.054,0.074]; P<0.001, Trump supporters: AME =0.055; 95% CI=[0.044,0.066]; P<0.001). Replicating earlier work (4, 72–74), we further find that participants preferring Trump performed, on average, significantly worse at identifying misleading posts than participants preferring Biden (AME =−0.016; 95% CI=[−0.021,−0.011]; P=0.001). Also, participants were, on average, significantly more likely to correctly identify misleading posts that were politically discordant (AME =0.092; 95% CI=[0.087,0.097]; P<0.001) than those that were politically concordant (AME =−0.057; 95% CI=[−0.057,0.064]; P=0.001.

The advantage of community notes over simple expert flags in improving the identification of misleading posts depended on the political congruence of the post (see Fig. 4d). For Biden supporters, replacing an expert flag with a community note would have increased the perceived misleadingness by 2.4 percentage points for politically concordant posts (AME =0.024; 95% CI=[0.007,0.039]; P=0.009), and by 4.5 percentage points for politically neutral posts (AME =0.045; 95% CI=[0.031,0.059]; P<0.001). We observe no statistically significant effect for politically discordant posts (AME =0.005; 95% CI=[−0.007,0.016]; P=0.439). For Trump supporters, replacing an expert flag with a community note would have increased the perceived misleadingness by 4.5 percentage points for politically concordant posts (AME =0.045; 95% CI=[0.027,0.065]; P<0.001), and by 4.4 percentage points for politically neutral posts (AME =0.044; 95% CI=[0.026,0.062]; P<0.001). For politically discordant posts, the effect was not statistically significant (AME =−0.012; 95% CI=[−0.027,0.003]; P=0.112). Hence, both Biden supporters and Trump supporters were relatively more successful in identifying misinformation if exposed to community notes for politically concordant or neutral posts, but not for politically discordant posts.

Across all fact-checking interventions, there were significant treatment condition effects on untagged (i.e. nonlabeled) nonmisleading posts (see Fig. 4b); that is, we observed an “implied truth” effect on untagged posts (28). Compared to the control condition (in which no fact-check was shown), displaying fact-checks on misleading posts decreased the perceived misleadingness of untagged nonmisleading post by, on average, 5.2 percentage points for expert flags (AME =−0.052; 95% CI=[−0.059,−0.044]; P=0.001), 5.1 percentage points for community flags (AME =−0.051; 95% CI=[−0.058,−0.043]; P=0.001), and 3.2 percentage points for community notes (AME =−0.032; 95% CI=[−0.039,−0.025]; P=0.001). Hence, the treatment condition effect on untagged nonmisleading posts was significantly less pronounced for community notes than for simple misinformation flags (each P≤0.001). There were no statistically significant variations in these effects for Biden vs. Trump supporters (P=0.306 for expert flags, P=0.587 for community flags, P=0.214 for community notes).

Altogether, these results imply that community notes consistently improved the identification of misleading posts over simple misinformation flags. However, simple misinformation flags increased belief in untagged, nonmisleading posts to a larger extent than community notes. As a result, when comparing the aggregated average marginal effects across both misleading and nonmisleading posts (i.e. overall discernment), the advantage of community notes over simple misinformation flags was small (see Fig. 4c). Compared to the control condition, displaying fact-checks on misleading posts increased the overall discernment of misleading vs. nonmisleading posts by, on average, 6.1 percentage points for expert flags (AME =0.061; 95% CI=[0.056,0.066]; P<0.001), 5.5 percentage points for community flags (AME =0.055; 95% CI=[0.050,0.060]; P<0.001), and 6.4 percentage points for community notes (AME =0.064; 95% CI=[0.059,0.069]; P<0.001). The differences between these average marginal effects were statistically significant for expert flags vs. community flags (P=0.021) and community notes vs. community flags P<0.001); however, not for community notes vs. expert flags (P=0.334).

### Demographics, beliefs, and cognitive reflection

**Fig. 5.**
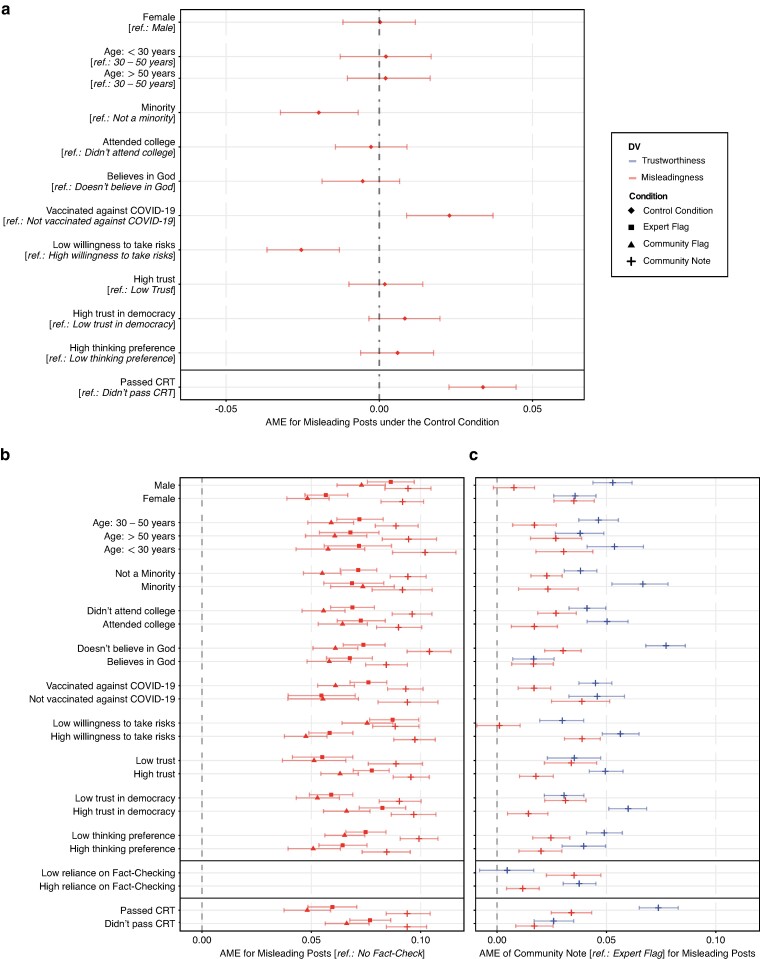
Effects of demographics, fact-checking reliance, and cognitive reflection. a) Average marginal effects (AME) for misleading posts under the control condition. b) AMEs for misleading posts across different fact-checking conditions (relative to the control condition). c) AMEs when replacing an expert flag with a community note. AMEs (symbols) were calculated based on hierarchical linear regression models with interaction terms across two dependent variables (DV): trustworthiness, and misleadingness. The 7-point Likert-scale responses were rescaled to the interval [0, 1]. Control variables and random intercepts for posts and subjects were included. The 95% confidence intervals (error bars) were derived using the bootstrap method for 1,000 resamples. Full results are in Tables S10 to S16.

We examined differences across demographics and participants’ beliefs towards various topics, which we also asked about as part of our survey (see Materials and methods section). Furthermore, participants had to complete a 4-item Cognitive Reflection Test to measure their level of reflective thinking (75, 76) and those in the treatment conditions were additionally asked how they would rate their general reliance on fact-checks. The average marginal effects of these variables on participants’ trust in fact-checks and perceived misleadingness are visualized in Fig. 5. Full results and descriptive statistics are in Supplementary Material C and Table S1.

Figure 5a shows that under the control condition (in which no fact-check was shown), participants performed better in correctly identifying misleading posts if they were vaccinated against COVID-19 (AME =0.023; 95% CI=[0.009,0.037]; P=0.004) or have passed the CRT (AME =0.034; 95% CI=[0.023,0.045]; P<0.001). In contrast, participants were less accurate in identifying misleading posts if they belonged to an ethnical minority (AME =−0.020; 95% CI=[−0.032,−0.007]; P=0.007) or indicated a low willingness to take risks (AME =−0.025; 95% CI=[−0.037,−0.013]; P=0.001). We do not observe statistically significant links between the identification of misleading posts and other variables (see Fig. 5a). Overall, these results align well with previous work examining predictors of susceptibility to misinformation (77).

We further studied moderation effects, i.e. how the efficacy of fact-checking interventions varied depending on demographics, beliefs, and cognitive reflection (Fig. 5b). Confirming previous work (39), all considered fact-checking interventions had a broad efficacy. Across all conditions, belief in misinformation was consistently reduced across all demographics, i.e. misleading posts were more likely to be correctly identified (each P<0.001).

When comparing the efficacy of different fact-checking interventions (Fig. 5c), replacing an expert flag with a community note would have consistently increased trust in fact-checks (each P<0.001); except for participants who indicated that their general reliance on fact-checks is low (AME =0.005; 95% CI=[−0.008,0.017]; P=0.439). The advantage of community notes over expert flags was particularly pronounced for male (AME =0.053; 95% CI=[0.044,0.062]; P<0.001) and young participants (AME =0.054; 95% CI=[0.041,0.067]; P<0.001), ethnic minorities (AME =0.067; 95% CI=[0.053,0.078]; P<0.001), participants who stated that they did not believe in God(s) (AME =0.077; 95% CI=[0.068,0.086]; P<0.001), and those who passed the CRT (AME =0.074; 95% CI=[0.065,0.083]; P<0.001). Regarding the identification of misleading posts, the highest improvements were observed for female participants (AME =0.035; 95% CI=[0.026,0.044]; P<0.001), participants who were not vaccinated against COVID-19 (AME =0.039; 95% CI=[0.025,0.051]; P<0.001), and for participants who indicated having a high willingness to take risks (AME =0.039; 95% CI=[0.031,0.047]; P<0.001).

### Effect of presentation format and context

**Fig. 6.**
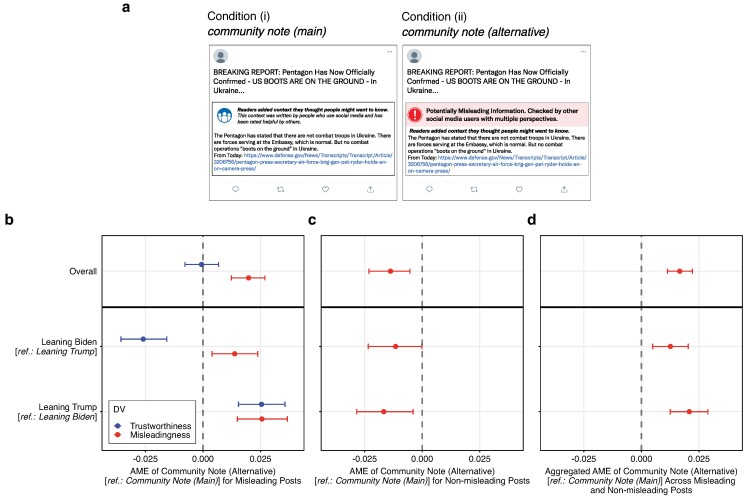
Additional experiment for effects of presentation format. a) Example of a social media post and fact-checks shown to participants. Participants were randomly assigned to one of two conditions: (i) *community note (main)*, where misleading posts were supplemented with a textual community note without an explicit warning label (i.e. previous condition 4), or (ii) *community note (alternative)*, where misleading posts were supplemented by a combination of a textual community note and an explicit warning label indicating that community fact-checkers categorized the content as being misleading. Posts were politically balanced to appeal to subjects with different political views (pro-Republican, pro-Democrat, and politically neutral). b) Average marginal effects (AME) and 95% confidence intervals from a hierarchical linear regression model with interaction terms predicting trustworthiness, and misleadingness (7-point Likert scale normalized to the interval [0, 1]) for misleading posts. c) AMEs for nonmisleading posts. d) Aggregated AMEs for misleading and nonmisleading posts (i.e. overall discernment). The 95% confidence intervals (error bars) were derived using the bootstrap method for 1,000 resamples. N= 12.168 (24.300) observations across 675 participants. Full results are in Tables S22 and S23.

We carried out an additional experiment to study the extent to which different presentation formats influence the perceived trustworthiness of community notes. In the additional experiment, n=675 participants ($M_{\mathrm{age}}=43$, 51% female, politically balanced) were randomly assigned to one of two conditions, representing two different presentation formats of community notes (see Fig. 6a). The two formats differed in such a way that the textual community note was presented either without an explicit warning label (i.e. former condition 4) or with an explicit warning label indicating that community fact-checkers categorized the content as being misleading (i.e. a combination of former conditions 3 and 4). Apart from the treatments, the experiment had the same elements and was conducted in exactly the same way as the original experiment (see Materials and methods section).

We fitted hierarchical linear regression models to quantify the effects of the alternative presentation format of community notes on trust in fact-checks and the identification of misleading and nonmisleading posts (see Supplementary Material C). Figure 6b or c visualizes the average marginal effects (AME). Regarding trust in fact-checks, there were, on average, no statistically significant differences between the two presentation formats (AME =−0.001; 95% CI=[−0.008,0.007]; P=0.846). However, there were significant differences when looking at Biden vs. Trump supporters individually. For Biden supporters, the alternative presentation format with an explicit warning label decreased the trust in community notes by 2.6 percentage points (AME =−0.026; 95% CI=[−0.036,−0.016]; P=0.001). In contrast, the alternative presentation format increased trust in community notes by 2.5 percentage points for participants leaning towards Trump (AME =0.025; 95% CI=[0.015,0.036]; P<0.001).

We linearly aggregated the effect sizes from both experiments to provide an estimate for the isolated effect of adding context to community fact-checks (i.e. warning label vs. warning label + context). Compared to context-free community warning labels (i.e. condition 3 in the main experiment), community notes combining the same warning label with context (i.e. the alternative presentation format in the additional experiment) were, on average, linked to 5.35 percentage points higher trust in fact-checks (95% CI=[0.047,0.060]; P<0.001). In terms of percentages, this translates to an increase in trust of 9.35%. Notably, the isolated effect of fact-checking context on trust was larger for Trump supporters with 6.65 percentage points (95%CI=[0.057,0.076]; P<0.001) than for Biden supporters with 3.47 percentage points (95% CI=[0.027,0.044]; P<0.001).

The alternative presentation format improved the identification of misleading and nonmisleading posts. On average, presenting community notes with an explicit warning label increased the perceived misleadingness of misleading posts by 2.0 percentage points (AME =0.020; 95% CI=[0.012,0.027]; P<0.001), and decreased the perceived misleadingness of nonmisleading posts by 1.4 percentage points (AME =−0.014; 95% CI=[−0.023,−0.005]; P=0.001). The variation across the political leanings of the participants was small. For Biden supporters, we observe a smaller positive effect for misleading posts (AME =0.014; 95% CI=[0.004,0.024]; P=0.011) and no statistically significant effect for nonmisleading posts (AME =−0.012; 95% CI=[−0.023,0.000]; P=0.050). For Trump supporters, we observe a slightly larger positive effect for misleading posts (AME =0.026; 95% CI=[0.015,0.037]; P<0.001) and a slightly larger negative effect for nonmisleading posts (AME =−0.017; 95% CI=[−0.028,−0.004]; P=0.011). In terms of overall discernment, community notes with an explicit warning label significantly increased participants’ accuracy by, on average, 1.66 percentage points (AME =0.017; 95% CI=[0.011,0.022]; P<0.001). This effect was again larger for Trump supporters (AME =0.021; 95% CI=[0.012,0.029]; P<0.001) than for Biden supporters (AME =0.013; 95% CI=[0.005,0.020]; P=0.003).

We again related these numbers back to the results from our main experiment to assess the isolated effect of context on the identification of misleading vs. nonmisleading posts. Compared to context-free community warning labels, adding context increased the perceived misleadingness of misleading by posts by 5.54 percentage points on average (95% CI=[0.049,0.062]; P<0.001). In terms of percentages, this number translates to an increase in perceived misleadingness of 6.99%. The increase was slightly higher for participants leaning towards Biden with 5.95 percentage points (95% CI=[0.051,0.068]; P<0.001) than for those leaning towards Trump with 5.07 percentage points (95% CI=[0.041,0.061]; P<0.001). For nonmisleading posts, the effect of additional context was small (0.53 percentage points overall; 0.37 percentage points for Biden supporters; and 0.70 percentage points for Trump supporters) and not statistically significant (P=0.155 overall; P=0.494 for Biden supporters; and P=0.234 for Trump supporters). The improvement in overall discernment of misleading vs. nonmisleading posts was 2.51 percentage points on average (95% CI=[0.020,0.030]; P<0.001), 2.78 percentage points for Biden supporters (95% CI=[0.021,0.034]; P<0.001), and 2.19 percentage points for Trump supporters (95% CI=[0.015,0.030]; P<0.001).

Overall, our additional experiment indicates that presentation effects for community notes were relatively small. We observed, on average, no statically significant differences in trustworthiness between community notes presenting solely context vs. community notes combining context with explicit warning labels. In combination with the findings from our main experiment, this further supports that the higher trustworthiness of community notes (vs. simple misinformation flags) primarily stems from the additional context rather than different formats of labels/warning messages. Nonetheless, combining fact-checking explanations with explicit warning labels may have the potential to slightly improve users’ ability to identify misinformation. A possible reason may be that the explicit warning labels offer users a more definite cue about whether or not a post is misleading, thereby encouraging them to adhere to the labels. In contrast, community notes that lack an explicit warning label and instead solely focus on offering context (i.e. a more “neutral” presentation) may imply to users that the judgment is, to a relatively larger extent, up to them. The consistent trust levels for community notes across both presentation formats indicate that, on average, these improvements may be achieved without compromising users’ trust in fact-checks.

## Discussion

Here, we presented n=1,810 Americans with 36 misleading and nonmisleading social media posts and assessed their trust in different types of fact-checking interventions. All posts represented real-world items from the social media platform X, and all fact-checks were surfaced by Community Notes’ bridging algorithm. Across all demographics and political leanings, we found that text-based community notes explaining why a fact-checked post was misleading were perceived as significantly more trustworthy than simple (i.e. context-free) misinformation flags. Only marginal differences were observed between simple expert flags and simple community flags. This implies that the increases in trustworthiness primarily stemmed from the context provided in community notes rather than the fact-checking source. Furthermore, in an additional experiment, we found no statically significant differences in trustworthiness across different presentation formats, that is, between community notes presenting solely text-based explanations vs. community notes combining warning labels and text-based explanations. Overall, our findings demonstrate that context matters in fact-checking on social media and that exposing users to text-based community notes might be an effective approach to mitigate trust issues with simple misinformation flags.

Previous work has evaluated the efficacy of simple misinformation flags regarding misinformation discernment and sharing intentions (23–30, 32). Consistent with the vast majority of these works, we find that fact-checking interventions significantly improved the identification of misleading and nonmisleading social media posts (we also analyzed sharing intentions; yet, we relegated these findings to the Supplementary material due to potential spillover effects when asking participants about misleadingness before assessing sharing intentions (78); see Supplementary Material C.1). However, the advantage of community notes (over simple misinformation flags) depended on the political congruence of the fact-checked post. Across both sides of the political spectrum, replacing a simple misinformation flag with a community note would have made users’ assessments relatively more accurate for politically concordant misinformation, but not for politically discordant misinformation. Hence, the context in community notes appears to be particularly helpful in countering resistance to fact-checking for partisan-aligned misinformation (e.g. a Trump supporter exposed to COVID-19 misinformation). This is an encouraging finding, as politically concordant misleading posts are also the type of misinformation that people are more likely to believe and share on social media. In contrast, additional context might be (relatively) less helpful for politically discordant misinformation (e.g. a Biden supporter exposed to COVID-19 misinformation), which users might perceive as clearly false and which might result in them being more inclined to rely on their existing beliefs or knowledge. Interestingly, our analysis further showed that treatment condition effects on untagged nonmisleading posts were significantly less pronounced for community notes than for simple misinformation flags. This indicates that the presence of community notes on misleading posts led to a less strong expectation that untagged, nonmisleading posts were true.

Our findings reinforce the notion that partisanship plays a key role regarding the success of fact-checking interventions (22). While the majority of users across both sides of the political spectrum trusted the fact-checks in our study, participants preferring Trump over Biden were overall significantly less likely to do so. In particular, Trump supporters tended to distrust fact-checking context for politically concordant misinformation such as, for example, COVID-19 misinformation. These observations align with prior studies, suggesting that adherents to the left tend to be less tolerable to the spread of misinformation and have greater trust in fact-checking (37, 38). Moreover, fact-checks were, on average, perceived as less trustworthy if the misleading post was politically concordant. This observation is supported by prior research that humans seek information that confirms their partisan preferences (79) and that the perceived credibility of information is greater when it is consistent with the recipient’s existing political beliefs (80, 81). Replicating earlier findings (4, 72–74), we also observed that participants leaning to the political right were less accurate in identifying misleading posts, and more likely to re-share misinformation.

As with other research, ours is not free of limitations that offer opportunities for future research. First, analogous to earlier works (28, 30, 45), only posts that were verifiably misleading were presented with fact-checks and *all* misleading posts in the treatment conditions received flags/community notes. In practice, however, the validity of misinformation warnings can vary, and invalid misinformation warnings may lead individuals to discard authentic content (82). Thus, future studies should examine how users’ behavior changes if presented with erroneously labeled posts and the effects of labeling varying proportions of misleading posts. Second, our study focused on the cultural context of American misinformation and American participants. While previous research suggests that solutions to the misinformation challenge may be similarly effective around the globe (77), future work should nonetheless explore how our findings translate to other countries and cultural contexts. Third, the survey participants recruited via Prolific were not nationally representative. Although our study was conducted among social media users, the survey population likely overrepresented higher-educated users. Fourth, additional limitations arise due to the selection of post and (high-quality) fact-checks surfaced by Community Notes’ bridging algorithm. While we ensured that posts were politically balanced to appeal to subjects with different political views, more research is necessary to understand how the efficacy varies for community notes of varying quality, writing styles, and political stances. Fifth, future research may experiment with additional formats for the design of flags and notes. However, our study indicates that such presentation effects may be relatively small. Ultimately, our work was performed in an experimental context, which differs from the common environment users face when browsing through social media. However, the use of online surveys provided a sandbox to test otherwise challenging interventions, and previous works have shown that misinformation interventions replicate well in field experiments (83).

From a broader perspective, misinformation on social media poses serious threats to democracy and society as a whole. Major social media providers thus have been called upon to develop effective countermeasures to combat the spread of misinformation on their platforms. However, current approaches to fact-checking social media content do not live up to their full potential. A major challenge is that fact-checking on social media must deal with distrust towards fact-checkers. Our research demonstrates that community-based fact-checking systems (e.g. X’s Community Notes) that focus on providing fact-checking context have the potential to at least mitigate trust issues that are common in traditional approaches to fact-checking. Fostering trust in fact-checking is vitally important, especially as we face upcoming events, such as elections, and emerging challenges due to the scalability of AI-generated misinformation.

## Materials and methods

### Participants

We recruited a large sample of American residents (total $n=1,810,M_{\mathrm{age}}=42$ years, 51% female, 52% preferred Biden over Trump) via Prolific (https://www.prolific.co/) across seven experimental sessions conducted between April and June 2023 (see Supplementary Material D for details). The study design was identical in all sessions. Although Prolific is not nationally representative, research shows that it provides high-quality data (84).

To achieve a representative distribution of Trump and Biden supporters (Republicans are underrepresented on Prolific (84)), we explicitly recruited Biden and Trump supporters, respectively, in sessions 2–7. To do so, we used the prescreening tool available through Prolific, contacting only participants who indicated they voted for Trump or Biden in the 2020 presidential election. Furthermore, we ensured that the participants were representative across genders in all sessions (women are overrepresented on Prolific). Detailed summary statistics are in Supplementary Material A.

### Materials

All participants were presented with the same set of 36 social media posts (18 “misleading” and 18 “nonmisleading” posts) across multiple topics (e.g. Politics, Business, Health, Celebrities). All posts were presented in a standard “X format” (see example in Fig. 1). To minimize author-specific effects, the posts were anonymized, and all information about likes, shares, and comments was removed. The misleading social media posts and corresponding fact-checks have been manually selected from X’s Community notes platform (available via https://twitter.com/i/communitynotes). At the time of selection, each fact-check has been classified as helpful by Community Notes’ bridging algorithm, i.e. was rated helpful by multiple users with diverse viewpoints (54) (see Supplementary Material E). The nonmisleading social media posts have been manually selected from X to cover similar stories and topics. Hence, all fact-checks and posts in our study represent real-world items from a major social media platform. All posts were additionally fact-checked by three trained research assistants who accessed professional fact-checks (snopes.com, factcheck.org, etc.) and other reliable sources to ensure that the labels for misleading and nonmisleading post items were accurate (see Supplementary Material E).

The post items in the misleading and nonmisleading categories were politically balanced to appeal to subjects with different political views (see Supplementary Material E). Specifically, in each category, six posts were Republican-consistent (pro-Republican/anti-Democrat), six were Democrat-consistent (pro-Democrat/anti-Republican), and six were politically neutral. To examine differences in political orientation, pro-Democrat/anti-Republican posts were classified as concordant for participants favoring Biden over Trump and discordant for participants favoring Trump over Biden (and vice versa for pro-Republican/anti-Democrat posts). Politically neutral posts were classified as neutral for both groups of participants. The categorizations of the political orientations of the posts were validated with the help of three trained research assistants (see Supplementary Material E).

### Procedure

The procedure was identical in all experimental sessions. Participants were randomly assigned to one of four conditions (see Fig. 1) using a between-subject design: (1) *Control*, where the participants were presented only with the social media posts (i.e. without fact-checks), (2) *expert flag*, where participants were presented with the same posts, but misleading posts were supplemented by a flag indicating that expert fact-checkers categorized the content as being misleading, (3) *community flag*, where participants were presented with the same posts, but misleading posts were supplemented by a flag indicating that community fact-checkers categorized the content as being misleading, or (4) *community note*, where participants were presented with or preparation of tthe same posts, but misleading posts were supplemented with textual community notes explaining why the information in the post is misleading. In conditions (2–4), all misleading posts have been supplemented by the respective fact-check, and all nonmisleading posts have been displayed without any fact-check. Posts were displayed to participants in a random order to minimize presentation-order effects.

Before starting the survey, participants were presented with the following instructions: “In this survey, you will be presented with a set of social media posts. Please read the posts carefully and answer the following questions for each post: (1) To the best of your knowledge, is the above social media post misleading? (2) How trustworthy do you think the [expert fact-check, community fact-check, textual community note] for the above social media post is? (3) If you were to see the above content on social media, how likely would you be to share it?” Question 2 was only asked for misleading posts in conditions (2–4). Participants assigned to conditions (2–4) have been provided with a general explanation of the origin of the fact-checks. For each social media post, participants were asked to answer the above questions on a 7-point Likert-scale. The Likert scales for the three questions (Q1–Q3) were specified as follows: (Q1) Extremely Accurate (1) to Extremely Misleading (7); (Q2) Extremely Untrustworthy (1) to Extremely Trustworthy (7); (Q3) Extremely Unlikely (1) to Extremely Likely (7).

At the end of the survey, we asked participants questions about demographics, their beliefs toward various topics, and they had to answer a 4-item cognitive reflection test (see Supplementary Material D).

### Analysis

We implemented a hierarchical linear regression model with random intercepts for posts and subjects to predict participants’ trust in fact-checks at the level of the response item. For the sake of interpretability, we normalized the 7-point Likert scale responses to the interval [0, 1]. This allows us to interpret the effects in our model as percentage point changes. The key explanatory variables were dummies referring to the experimental conditions. For our analysis of trust in fact-checks, we restricted the analysis to misleading posts and conditions 2–4 (baseline = Condition 2/Expert flag). The reason is that the question regarding the trustworthiness of fact-checks was only asked when a fact-check was presented and thus only for misleading posts in conditions 2–4. In addition, we controlled for the political leaning of the participants, i.e. whether they favored Biden or Trump (baseline = Biden), and whether a specific post was aligned with a participant’s political leaning (concordant, neutral, or discordant; baseline = neutral). To examine interaction effects, we included three-way interaction terms between the condition dummies, the political leaning of the participants, and the political alignment of posts.

Additionally, we implemented hierarchical linear regression models to analyze how misinformation discernment varied across the four conditions. To this end, we used the misleadingness ratings for the posts as dependent variables. We again normalized the 7-point Likert scale responses to the interval [0, 1]. The key explanatory variables were dummies referring to the four experimental conditions (baseline = Condition 1/No fact-check), and a dummy indicating whether a post was nonmisleading (=1) or misleading (=0). The control variables for the political leanings of the participants and the political alignment of the posts were the same as in the previous model. Furthermore, we included four-way interaction terms between the condition dummies, the misleading dummy, the political leaning of the participants, and the political alignment of posts to study interaction effects. Random intercepts for posts and subjects were also included.

Since our models include multiple higher-order interactions, the coefficient estimates do not easily translate to our quantities of interest. Following best practices (85), we thus calculated the average marginal effects (AME) to interpret the effect sizes. AME is the difference in the average predicted effect between the group of interest and the reference group. For instance, an AME of +0.05 for the Trump dummy variable indicates a 5 percentage point difference in the expected value of the dependent variable for participants favoring Trump (as opposed to those favoring Biden).

Multiple exploratory analyses and checks validated our results and confirmed their robustness (see Supplementary Material C): (i) we analyzed how sharing intentions varied across the four condition. Furthermore, we performed additional moderation analyses (ii) to examine differences across demographics and participants’ beliefs towards various topics (e.g. belief in God, risk preferences); (iii) to examine how reliance on fact-checks is associated with participants’ ratings; (iv) to examine the role of cognitive reflection based on the outcomes of a 4-item Cognitive Reflection Test. (v) We conducted an additional experiment to test the effects of alternative presentation formats on the efficacy of community notes. As part of our robustness checks, we also (vi) tested a wide range of alternative model specifications. For instance, we repeated our analyses using logistic mixed-effects models treating the Likert-scale responses as binary variables, and repeated our analysis using a linear regression model with robust standard errors clustered on both subjects and posts. Moreover, we tested model variants including by-post and by-subject random slopes. However, these “maximal” models failed to converge or resulted in singular fits, i.e. overfitting. Random slopes were therefore omitted and we instead opted for models with crossed random intercepts (86). Finally, (vi) we performed a variety of model checks (e.g. assessing variance inflation factors) to ensure that our estimates are robust. In all cases, our results were robust and consistently supported our findings.

Our models were implemented in R 4.3.2 using the lme4 package and the marginaleffects package.

## Supplementary Material

pgae217_Supplementary_Data

## Data Availability

Code and anonymized data to replicate the results of this study are available through the Open Science Framework (OSF), https://osf.io/26msx/.
