# Supplementary material for: Community notes increase trust in fact-checking on social media
Source: PNAS Nexus. 2024 May 31;3(7):pgae217. doi: 10.1093/pnasnexus/pgae217 (PMC11212665; doi:10.1093/pnasnexus/pgae217)
Supplement: pgae217_Supplementary_Data [file pgae217_supplementary_data.pdf]

Supplementary Information for

Community notes increase trust in fact-checking on social media

Chiara Patricia Drolsbach, Kirill Solovev, and Nicolas Pröllochs

Corresponding Author: Nicolas Pröllochs

E-mail: nicolas.proellochs@wi.jlug.de

Contents

A Descriptive statistics **2**
A.1 Dependent variables . . . 2
A.2 Demographics, beliefs, and CRT . . . 3
A.3 Demographics, beliefs, and CRT of Trump vs. Biden supporters . . . 6
A.4 Attrition . . . 7
A.5 Social media behavior . . . 8
A.6 Perception of fact-checks . . . 10

B Estimation results **11**

C Additional analyses **15**
C.1 Sharing intentions . . . 15
C.2 Demographics and beliefs . . . 18
C.3 Reliance on fact-checks . . . 23
C.4 Cognitive Reflection Test (CRT) . . . 24
C.5 Analysis with hierarchical logistic regression models . . . 25
C.6 Analysis with cluster-robust standard errors . . . 28
C.7 Additional experiment for effects of presentation format . . . 32

D Further methodological details **35**
D.1 Participants . . . 35
D.2 Participants in additional experiment . . . 36
D.3 Additional question items . . . 36

E Selection of social media posts and fact-checks **39**

A. Descriptive statistics

A.1. Dependent variables. Figure S1 shows the distribution of the participants' responses to questions on trust in fact-checks (Figure S1a), misleadingness of posts (Figure S1b), and sharing intentions (Figure S1c) on 7-point Likert scales.

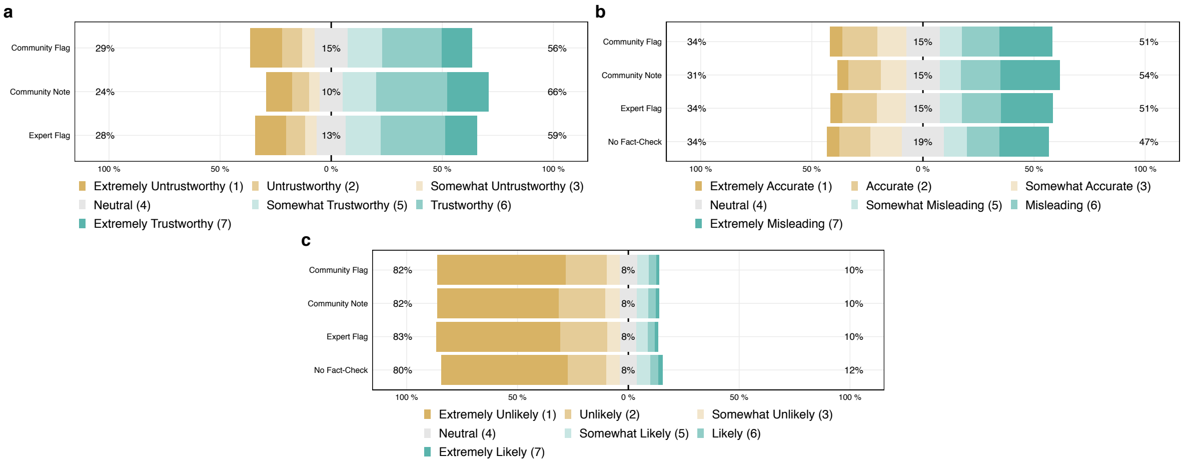

Fig. S1. Distribution of participants' responses across experimental conditions on 7-point Likert scales. (a) Trustworthiness of the fact-check. (b) Misleadingness of the post. (c) Sharing intentions.

A.2. Demographics, beliefs, and CRT. Table S1 shows the frequencies of the responses to questions on demographics and beliefs. Evidently, the variables are similarly distributed across the experimental conditions. Overall, 51 % of all participants were female, 48 % male, and 1.4 % identify themselves as non-binary. On average, the participants in our study were 42 years old and the majority of all participants did at least attend college (83 %). Out of all participants, 97 % indicated to be native English speakers, 75 % belong to an ethnic majority, and 74 % are vaccinated against COVID-19. Furthermore, more than half of all participants indicated that they believe in God (54 %). Our sample was approximately balanced across political leanings. Overall, 43 % of participants identified as Democrats and 38 % as Republican (19.7 % as third party or other). During the 2020 presidency election, 48 % indicated that they voted for Biden, whereas 45 % voted for Trump. When being forced to choose between Biden an Trump, 52 % prefer Biden as president and 48 % prefer Trump.

Subjects in our study were asked a series of questions regarding their attitude towards risk, trust, and preference for analytical thinking on a 5-point Likert scale. In the median, survey participants were "undecided" on their attitude towards risk ($Median_{Risk} = 3$) and trust in democracy ($Median_{TrustinDem} = 3$), had "somewhat" trust in general ($Median_{Trust} = 4$), and did "not really" prefer doing something that requires little thought over something that is challenging ($Median_{ThinkingPreference} = 2$).

Subjects in our study were also asked to complete a 4-item Cognitive Reflection Test (CRT). Overall, 43 % passed the CRT, which means that they answered all 4 questions correctly. Notably, this share is comparatively high, pointing towards a high-quality sample of participants from Prolific.

Table S1. Descriptive statistics of participants' responses to questions on demographics, beliefs, and CRT. Values are reported as frequencies and percentages, unless otherwise stated.

**Variable**	**Overall**	**No Fact-Check**	**Expert Flag**	**Community Flag**	**Community Note**
**Participants (n)**	1,810	463	448	422	477
**Gender**					
Female	923 (51%)	252 (54%)	228 (51%)	215 (51%)	228 (48%)
Male	861 (48%)	207 (45%)	215 (48%)	201 (48%)	238 (50%)
Non-binary	26 (1.4%)	4 (0.9%)	5 (1.1%)	6 (1.4%)	11 (2.3%)
**Age**					
Mean	42.00	42.41	41.62	42.13	41.84
**Level of Education**					
None	1 (<0.1%)	0 (0%)	1 (0.2%)	0 (0%)	0 (0%)
Less than high school degree	17 (0.9%)	2 (0.4%)	4 (0.9%)	6 (1.4%)	5 (1.0%)
High school diploma	272 (15%)	54 (12%)	70 (16%)	62 (15%)	86 (18%)
Attended college	478 (26%)	114 (25%)	127 (28%)	126 (30%)	111 (23%)
Bachelor's degree	731 (40%)	209 (45%)	177 (40%)	159 (38%)	186 (39%)
Graduate degree	311 (17%)	84 (18%)	69 (15%)	69 (16%)	89 (19%)
**Proficiency in English**					
Beginner	1 (<0.1%)	0 (0%)	1 (0.2%)	0 (0%)	0 (0%)
Intermediate	7 (0.4%)	3 (0.6%)	2 (0.4%)	0 (0%)	2 (0.4%)
Advanced	55 (3.0%)	12 (2.6%)	16 (3.6%)	16 (3.8%)	11 (2.3%)
Native Speaker	1,747 (97%)	448 (97%)	429 (96%)	406 (96%)	464 (97%)
**Belief in God(s)**					
I believe in God	972 (54%)	254 (55%)	230 (51%)	228 (54%)	260 (55%)
I don't believe in God	365 (20%)	85 (18%)	96 (21%)	83 (20%)	101 (21%)
I don't know whether or not God exists	330 (18%)	88 (19%)	87 (19%)	74 (18%)	81 (17%)
I don't really take a stance on God	143 (7.9%)	36 (7.8%)	35 (7.8%)	37 (8.8%)	35 (7.3%)
**Vaccinated against COVID-19**					
Unvaccinated	472 (26%)	119 (26%)	120 (27%)	105 (25%)	128 (27%)
Vaccinated	1,338 (74%)	344 (74%)	328 (73%)	317 (75%)	349 (73%)
**Ethnicity**					
Ethnic Majority	1,359 (75%)	341 (74%)	328 (73%)	323 (77%)	367 (77%)
Ethnic Minority	451 (25%)	122 (26%)	120 (27%)	99 (23%)	110 (23%)
**Political Orientation**					
Democrat	778 (43%)	187 (40%)	205 (46%)	176 (42%)	210 (44%)
Republican	683 (38%)	192 (41%)	160 (36%)	146 (35%)	185 (39%)
Third Party	103 (5.7%)	30 (6.5%)	27 (6.0%)	27 (6.4%)	19 (4.0%)
Other	246 (14%)	54 (12%)	56 (13%)	73 (17%)	63 (13%)
**Vote in 2020**					
Trump	811 (45%)	216 (47%)	198 (44%)	184 (44%)	213 (45%)
Biden	866 (48%)	209 (45%)	225 (50%)	199 (47%)	233 (49%)
Other Candidate	17 (0.9%)	5 (1.1%)	1 (0.2%)	7 (1.7%)	4 (0.8%)
I did not vote for reasons outside my control	41 (2.3%)	11 (2.4%)	10 (2.2%)	12 (2.8%)	8 (1.7%)
I did not vote but I could have	64 (3.5%)	19 (4.1%)	12 (2.7%)	17 (4.0%)	16 (3.4%)
I did not vote out of protest	11 (0.6%)	3 (0.6%)	2 (0.4%)	3 (0.7%)	3 (0.6%)
**Leaning Biden or Trump**					
Biden	935 (52%)	230 (50%)	242 (54%)	217 (51%)	246 (52%)
Trump	875 (48%)	233 (50%)	206 (46%)	205 (49%)	231 (48%)
**Willingness to take risks**					
Median (IQR)	3.00 (2.00, 4.00)	3.00 (2.00, 4.00)	3.00 (2.00, 4.00)	3.00 (2.00, 4.00)	3.00 (2.00, 4.00)
**Trust in general**					
Median (IQR)	4.00 (3.00, 4.00)	4.00 (3.00, 4.00)	4.00 (3.00, 4.00)	4.00 (3.00, 4.00)	4.00 (3.00, 4.00)
**Trust in Democracy**					
Median (IQR)	3.00 (2.00, 4.00)	3.00 (2.00, 4.00)	4.00 (2.00, 4.00)	3.00 (2.00, 4.00)	4.00 (2.00, 4.00)
**Thinking Preference**					
Median (IQR)	2.00 (2.00, 3.00)	2.00 (2.00, 3.00)	2.00 (2.00, 3.00)	2.00 (2.00, 3.00)	2.00 (2.00, 3.00)
**Cognitive Reflection Test**					
Failed	1,029 (57%)	281 (61%)	256 (57%)	230 (55%)	262 (55%)

Passed	781 (43%)	182 (39%)	192 (43%)	192 (45%)	215 (45%)
**General Reliance on Fact-Checks**					
Low	886 (66%)	0 (0%)	297 (66%)	324 (77%)	265 (56%)
High	461 (34%)	0 (0%)	151 (34%)	98 (23%)	212 (44%)

A.3. Demographics, beliefs, and CRT of Trump vs. Biden supporters. To assess representativeness beyond gender and political orientation, we looked at how other variables (demographics, beliefs, etc.) differed between participants preferring Trump vs. Biden. Figure S2 visualizes the distributions of participants' responses for relevant variables.

We observe that the proportion of female participants is about the same in both groups, pro-Democrats and pro-Republicans (52% vs. 51.5%). At the same time, the share of participants older than 40 years is clearly smaller among pro-Democrats (43.7% vs. 60.5%). Further, we find that participants who favored Biden were slightly less likely to be native English speakers (95.5% vs. 97.5%), more often belonged to ethnic minorities (28.7 % vs. 20.9%), and were less likely to believe in God (35.4% vs. 74%) compared to those who supported Trump. A larger portion of participants leaning towards Biden (vs. those leaning towards Trump) possessed academic degrees (53% vs. 62%), received the COVID-19 vaccine (91.8% vs. 54.8%), and reported a high reliance on fact-checks (42% vs. 26.2%). Additionally, participants preferring Biden were more successful in passing the CRT than those preferring Trump (38.9% vs. 47.4%).

Note that these observations align well with expectations. For instance, analysis of voter demographics in various recent US elections revealed that younger people, ethnic minorities, and individuals holding academic degrees were more likely to support the Democratic Party than the Republican Party, while those identifying as religious were less prevalent among Democratic voters (1, 2). Similarly, studies on attitudes towards COVID-19 vaccination showed that Democrats were more inclined to get vaccinated (3, 4). Moreover, distrust of fact-checkers was shown to be more widespread among pro-Republicans than among pro-Democrats, as pro-Republicans were more likely to perceive fact-checkers as biased (5).

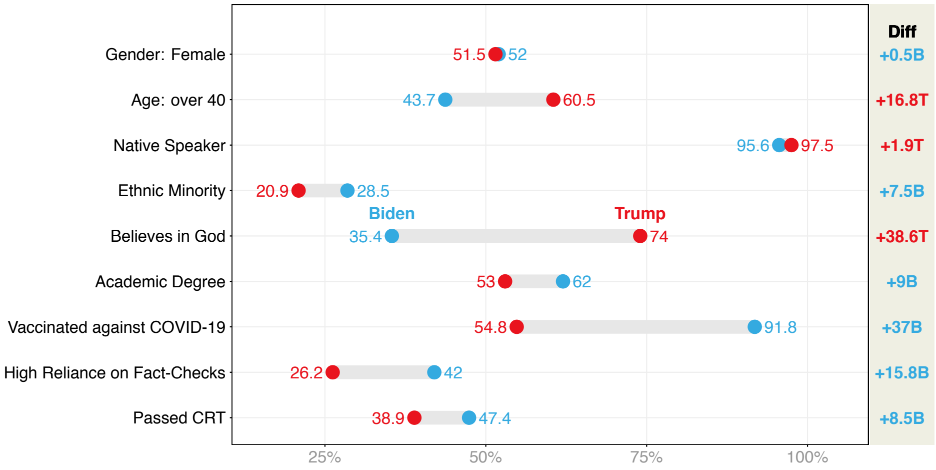

Fig. S2. Difference in participants' responses to questions on demographics, beliefs, and CRT, depending on their political leaning. The values show the percentage share of pro-Republican/pro-Trump (red) and pro-Democrat/pro-Biden (blue) participants in the respective group. The column "Diff" reports the difference between both groups, "T" and "B" stand for Trump and Biden.

A.4. Attrition. Before conducting our analysis, we adjusted the dataset to exclude participants who either did not complete the survey in full, indicated they had responded randomly, engaged in online research during the survey, or indicated they do not use social media. Additionally, those who failed the attention check were removed (see SI, Section D). Table S2 provides an overview of the number of participants excluded on the basis of these criteria.

We find that the variation in participant numbers across different conditions post-cleanup (see *Participants (after cleaning)* in Table S2) primarily reflects differences in the initial populations of these conditions (see *Participants (before cleaning)* in Table S2), i.e., randomness in the assignment via the survey tool. The share of participants excluded on the basis of the individual criteria was fairly consistent, and there were no statistically significant differences in the share of participants who completed the survey across the different conditions (Chi-square test: $\chi^2 = 9$; $P = 0.2133$, two-tailed). Overall, this suggests that attrition did not significantly affect the sizes of the treatment groups in our study.

Table S2. Distribution of different exclusion criteria per condition.

Variable	Overall	No Fact-Check	Expert Flag	Community Flag	Community Note
**Participants (before cleaning)**	2102	530	517	503	552
**Participants (after cleaning)**	1,810 (86.11%)	463 (87.35%)	448 (86.65%)	422 (83.90%)	477 (86.41%)
**Completed Survey**					
Yes	2014 (95.81%)	511 (96.42%)	492 (95.16%)	484 (96.22%)	527 (95.47%)
No	88 (4.19%)	19 (3.58%)	25 (4.84%)	19 (3.78%)	25 (4.53%)
**Answered Randomly**					
Yes	27 (1.19%)	6 (1.13%)	7 (1%)	5 (1%)	6 (0.5%)
No	1989 (94.62%)	505 (95.20%)	485 (94%)	479 (95%)	522 (94.5%)
N/A (didn't complete)	88 (4.19%)	19 (3.58%)	25 (4.84%)	19 (3.78%)	25 (4.53%)
**Searched Online**					
Yes	26 (1.14%)	5 (0.95%)	6 (1.16%)	5 (1.00%)	10 (1.81%)
No	1990 (94.67%)	506 (95.47%)	486 (94.00%)	479 (95.22%)	517 (93.66%)
N/A (didn't complete)	88 (4.19%)	19 (3.58%))	25 (4.84%)	19 (3.78%)	25 (4.53%)
**Social Media Account**					
Yes	1966 (93.53%)	500 (94.34%)	476 (92.07%)	473 (94.04%)	517 (93.66%)
No	48 (2.28%)	11 (2.08%)	16 (3.09%)	11 (2.18%)	10 (1.81%)
N/A (didn't complete)	88 (4.19%)	19 (3.58%))	25 (4.84%)	19 (3.78%)	25 (4.53%)
**Passed attention check**					
Yes	1900 (90.39%)	483 (91.13%)	472 (91.30%)	445 (88.47%)	500 (90.58%)
No	114 (5.42%)	28 (5.29%)	20 (3.86%)	39 (7.75%)	27 (4.89%)
N/A (didn't complete)	88 (4.19%)	19 (3.58%)	25 (4.84%)	19 (3.78%)	25 (4.53%)

A.5. Social media behavior. Table S3 reports the participants responses to questions regarding their social media behavior. 79 % of all participants use Facebook, 64 % use Twitter/X, and 68 % use Instagram. Prior to all analyses, all participants not using social media platforms were removed. Participants indicated that they are more likely to share content on political (41 %) and scientific (55 %) topics than on other topics. Out of all participants, 19 % stated that they do not share any content on social media. Participants where also asked to rate whether specific post characteristics are important for them when deciding about sharing a post. While accuracy and interestingness were extremely or very important to a majority of participants (91 % and 81 %), it was less important whether a post is funny (41 %) or surprising (22.5 %). Moreover, most participants indicated that it is at least moderately important for them that a post aligns with their beliefs (83 %). The participants' ratings on the importance of specific post characteristics did not differ drastically across different social media platforms and topics.

Table S3. Percentage of participants reporting having a social media account and type of content shared. Rows show participants' ratings of whether specific post characteristics are important for them when deciding about sharing a post.

	Social media outlets used							Type of content shared							
**Variable**	**Facebook**	**Twitter**	**Snapchat**	**Instagram**	**WhatsApp**	**TikTok**	**Other**	**Politics**	**Sports**	**Celebrities**	**Science**	**Business**	**Other**	**None**
**Participants (n)**	1,441	1,156	486	1,227	346	721	423	741	582	454	989	479	455	344
**Post is Accurate**														
Not at all	2.2%	1.5%	1.4%	2.0%	1.4%	1.1%	3.3%	0.5%	0.3%	0.7%	0.5%	0.2%	0.9%	8.4%
Slightly	0.9%	1.4%	1.2%	1.4%	0.3%	1.7%	0.7%	1.2%	1.4%	2.2%	1.4%	1.3%	1.3%	1.5%
Moderately	6.5%	7.8%	8.4%	7.7%	6.9%	7.6%	5.9%	8.1%	7.6%	8.1%	7.0%	7.5%	7.9%	5.2%
Very	26%	29%	29%	27%	27%	27%	25%	31%	32%	32%	30%	32%	26%	16%
Extremely	65%	61%	60%	62%	65%	63%	65%	59%	58%	57%	61%	59%	64%	69%
**Post is Surprising**														
Not at all	26%	24%	22%	25%	21%	22%	30%	18%	18%	12%	19%	18%	31%	46%
Slightly	22%	24%	26%	22%	16%	24%	24%	24%	23%	21%	24%	22%	27%	18%
Moderately	30%	32%	28%	31%	32%	31%	30%	32%	33%	33%	34%	32%	29%	22%
Very	16%	15%	17%	15%	22%	17%	13%	19%	21%	26%	17%	22%	9.5%	6.4%
Extremely	6.5%	5.5%	7.4%	6.2%	9.5%	6.4%	3.5%	6.6%	5.7%	7.5%	5.7%	6.1%	3.3%	8.4%
**Post is Interesting**														
Not at all	2.8%	1.6%	1.6%	2.3%	4.0%	1.2%	4.7%	0.3%	0.5%	0.2%	0.3%	0 (0%)	1.8%	12%
Slightly	2.2%	2.2%	1.9%	2.8%	2.0%	2.5%	3.5%	2.2%	1.7%	1.8%	1.9%	1.5%	2.2%	3.5%
Moderately	13%	14%	17%	14%	14%	13%	13%	13%	13%	10%	13%	14%	15%	14%
Very	43%	44%	42%	43%	43%	44%	43%	47%	47%	47%	46%	47%	45%	30%
Extremely	39%	38%	37%	38%	37%	40%	35%	38%	38%	41%	39%	38%	37%	40%
**Post is Aligned to the User's Beliefs**														
Not at all	8.1%	8.2%	6.8%	8.1%	7.2%	6.4%	11%	4.7%	4.1%	4.8%	6.2%	4.6%	7.0%	19%
Slightly	8.1%	10%	9.3%	9.4%	6.6%	8.9%	11%	9.3%	12%	9.0%	12%	11%	8.6%	7.8%
Moderately	23%	24%	23%	22%	21%	23%	26%	26%	27%	25%	27%	31%	25%	15%
Very	30%	30%	32%	31%	33%	30%	30%	32%	32%	33%	30%	32%	31%	23%
Extremely	30%	27%	29%	30%	32%	31%	22%	28%	25%	29%	25%	23%	28%	34%
**Post is Funny**														
Not at all	11%	10%	8.4%	10%	6.9%	7.9%	13%	9.9%	6.7%	5.7%	8.3%	9.2%	9.9%	18%
Slightly	16%	18%	15%	18%	18%	15%	18%	20%	17%	15%	20%	19%	16%	13%
Moderately	31%	32%	30%	31%	29%	31%	34%	34%	33%	31%	33%	33%	35%	26%
Very	22%	22%	28%	22%	27%	25%	20%	23%	26%	25%	23%	24%	19%	18%
Extremely	19%	18%	19%	19%	19%	20%	15%	14%	17%	23%	16%	15%	20%	24%

A.6. Perception of fact-checks. Table S4 reports the participants awareness of fact-checks and the influence the fact-checks had on their responses during the survey. Among participants in conditions 2 – 4, a vast majority (75 %) was aware of fact-checking prior to participating. However, awareness was substantially higher for expert fact-checks (92 %) than for community flags (65 %) and community notes (66 %). In general, participants' reliance on fact-checks differed a lot, but was similarly distributed across all conditions. Overall, 34 % indicated to have a high or extreme reliance on fact-checks.

Furthermore, participants were asked to indicate what influence the presented fact-check had on their discernment of posts with and without fact-checks during the survey. Overall, the majority of participants stated the the presence of fact-checks made them rate potentially misleading post as less accurate (52 %) or the tag had not influence (20 %). Posts without fact-checks (i.e., that were not potentially misleading) have been rated as more accurate (38.5 %) or the tag had no influence (43 %). Interestingly, the share of participants in condition 4 (community note) indicating that they rated posts with a community note (slightly) more accurate was much higher than in the other conditions (44 % versus 14.5 % and 14.3 %).

Table S4. Participants' perception of fact-checks.

Variable	Overall	No Fact-Check	Expert Flag	Community Flag	Community Note
Participants (n)	1,810	463	448	422	477
**Aware of Fact-Checking (Prior to Survey)**					
Not Aware of Fact-Checking	345 (26%)	–	37 (8.3%)	146 (35%)	162 (34%)
Aware of Fact-Checking	1,002 (74%)	–	411 (92%)	276 (65%)	315 (66%)
**Reliance on Fact-Checks**					
None (1)	193 (14%)	–	75 (17%)	76 (18%)	42 (8.8%)
Slight (2)	272 (20%)	–	94 (21%)	110 (26%)	68 (14%)
Moderate (3)	421 (31%)	–	128 (29%)	138 (33%)	155 (32%)
High (4)	328 (24%)	–	114 (25%)	67 (16%)	147 (31%)
Extreme (5)	133 (9.9%)	–	37 (8.3%)	31 (7.3%)	65 (14%)
**Influence of Fact-Checks (Misleading Posts)**					
Much less accurate (1)	223 (17%)	–	105 (23%)	61 (14%)	57 (12%)
Less accurate (2)	376 (28%)	–	148 (33%)	140 (33%)	88 (18%)
Slightly less accurate (3)	148 (11%)	–	52 (12%)	68 (16%)	28 (5.9%)
Tag had no influence (4)	274 (20%)	–	87 (19%)	97 (23%)	90 (19%)
Slightly more accurate (5)	179 (13%)	–	31 (6.9%)	37 (8.8%)	111 (23%)
More accurate (6)	118 (8.8%)	–	20 (4.5%)	16 (3.8%)	82 (17%)
Much more accurate (7)	29 (2.2%)	–	5 (1.1%)	3 (0.7%)	21 (4.4%)
**Influence of Fact-Checks (Non-misleading Posts)**					
Much less accurate (1)	36 (2.7%)	–	6 (1.3%)	14 (3.3%)	16 (3.4%)
Less accurate (2)	100 (7.4%)	–	16 (3.6%)	30 (7.1%)	54 (11%)
Slightly less accurate (3)	116 (8.6%)	–	23 (5.1%)	26 (6.2%)	67 (14%)
Tag had no influence (4)	575 (43%)	–	183 (41%)	183 (43%)	209 (44%)
Slightly more accurate (5)	366 (27%)	–	161 (36%)	118 (28%)	87 (18%)
More accurate (6)	122 (9.1%)	–	50 (11%)	38 (9.0%)	34 (7.1%)
Much more accurate (7)	32 (2.4%)	–	9 (2.0%)	13 (3.1%)	10 (2.1%)

B. Estimation results

To predict the participant's (i) trust in fact-checks and (ii) misleadingness ratings, we implemented a hierarchical linear regression model with random intercepts for posts and subjects. Average marginal effects (AMEs) and coefficient estimates are reported in Tables S5 and S7 (trust in fact-checks) and Tables S6 and S8 (misleadingness). The findings are described in detail in Section Results of the main paper.

Table S5. Average marginal effects (AME) and 95 % confidence intervals from a hierarchical linear regression model with three-way interaction terms predicting the trustworthiness of a fact-check (7-point Likert scale normalized to the interval [0, 1]). AME is the difference in the average predicted trustworthiness ratings between the group of interest and the reference group (e.g., an AME of $+0.05$ indicates a 5 percentage point difference in trustworthiness ratings). The indented rows report the AMEs of an intervention depending on the political leanings of participants (leaning Trump vs. Biden) and the political congruence of the fact-checked posts (concordant, neutral, discordant). Random intercepts for posts and subjects are included. The 95 % confidence intervals for the AMEs were derived using the bootstrap method for 1,000 resamples. N = 24,003 observations across 1,347 participants. Coefficient estimates are in SI, Table S7.

	AME	Lower CI	Upper CI	P-value
Community Note [*ref.: Expert Flag*]	0.048	0.042	0.055	< 0.001
Leaning Biden	0.061	0.052	0.069	< 0.001
Leaning Biden, *Concordant*	0.064	0.049	0.080	< 0.001
Leaning Biden, *Neutral*	0.074	0.061	0.086	< 0.001
Leaning Biden, *Discordant*	0.045	0.032	0.058	< 0.001
Leaning Trump	0.035	0.026	0.045	< 0.001
Leaning Trump, *Concordant*	0.023	0.007	0.039	0.010
Leaning Trump, *Neutral*	0.042	0.025	0.059	< 0.001
Leaning Trump, *Discordant*	0.040	0.024	0.056	< 0.001
Community Flag [*ref.: Expert Flag*]	–0.013	–0.020	–0.006	0.003
Leaning Biden	–0.010	–0.019	0.000	0.045
Leaning Biden, *Concordant*	–0.008	–0.027	0.009	0.391
Leaning Biden, *Neutral*	–0.010	–0.024	0.006	0.188
Leaning Biden, *Discordant*	–0.011	–0.026	0.003	0.155
Leaning Trump	–0.016	–0.026	–0.006	0.006
Leaning Trump, *Concordant*	–0.027	–0.044	–0.010	0.001
Leaning Trump, *Neutral*	–0.007	–0.025	0.011	0.441
Leaning Trump, *Discordant*	–0.014	–0.031	0.004	0.118
Leaning Trump [*ref.: Leaning Biden*]	–0.084	–0.089	–0.078	0.001
Concordant [*ref.: Neutral*]	–0.023	–0.029	–0.017	0.001
Leaning Biden	–0.034	–0.043	–0.026	0.001
Leaning Trump	–0.010	–0.020	0.000	0.040
Discordant [*ref.: Neutral*]	0.022	0.016	0.028	< 0.001
Leaning Biden	0.024	0.016	0.032	< 0.001
Leaning Trump	0.019	0.009	0.028	< 0.001

Table S6. Average marginal effects (AME) and 95 % confidence intervals from a hierarchical linear regression model with four-way interaction terms predicting the perceived misleadingness of a post (7-point Likert scale normalized to the interval [0, 1]). AME is the difference in the average predicted misleadingness ratings between the group of interest and the reference group (e.g., an AME of $+0.05$ indicates a 5 percentage point difference in misleadingness ratings). AMEs are reported separately for misleading (columns 2–5) and non-misleading posts (columns 6–9). The indented rows report the AMEs of an intervention depending on the political leanings of participants (leaning Trump vs. Biden) and the political congruence of the fact-checked posts (concordant, neutral, discordant). Random intercepts for posts and subjects are included. The 95 % confidence intervals for the AMEs were derived using the bootstrap method for 1,000 resamples. $N =$ 64,454 observations across 1,810 participants. Coefficient estimates are in SI, Table S8.

	Misleading				Non-misleading			
	**AME**	**Lower CI**	**Upper CI**	**P-value**	**AME**	**Lower CI**	**Upper CI**	**P-value**
Expert Flag [*ref.: No Fact-Check*]	0.071	0.063	0.078	< 0.001	–0.052	–0.059	–0.044	0.001
Leaning Biden	0.085	0.076	0.094	< 0.001	–0.057	–0.067	–0.048	0.001
Leaning Trump	0.055	0.044	0.067	< 0.001	–0.046	–0.056	–0.035	0.001
Community Flag [*ref.: No Fact-Check*]	0.060	0.053	0.067	< 0.001	–0.051	–0.058	–0.043	0.001
Leaning Biden	0.064	0.054	0.074	< 0.001	–0.053	–0.063	–0.044	0.001
Leaning Trump	0.055	0.044	0.066	< 0.001	–0.047	–0.058	–0.036	0.001
Community Note [*ref.: No Fact-Check*]	0.096	0.089	0.102	< 0.001	–0.032	–0.039	–0.025	0.001
Leaning Biden	0.110	0.100	0.120	< 0.001	–0.038	–0.048	–0.029	0.001
Leaning Trump	0.081	0.070	0.091	< 0.001	–0.024	–0.035	–0.014	0.001
Community Note [*ref.: Expert Flag*]	0.025	0.019	0.032	< 0.001	0.020	0.013	0.027	< 0.001
Leaning Biden, *Concordant*	0.024	0.007	0.039	0.009	0.014	–0.001	0.029	0.060
Leaning Biden, *Neutral*	0.045	0.031	0.059	< 0.001	0.016	0.001	0.032	0.042
Leaning Biden, *Discordant*	0.005	–0.007	0.016	0.439	0.027	0.008	0.046	0.003
Leaning Trump, *Concordant*	0.045	0.027	0.065	< 0.001	0.035	0.016	0.053	< 0.001
Leaning Trump, *Neutral*	0.044	0.026	0.062	< 0.001	0.019	0.003	0.036	0.020
Leaning Trump, *Discordant*	–0.012	–0.027	0.003	0.112	0.009	–0.011	0.029	0.385
Community Flag [*ref.: Expert Flag*]	–0.011	–0.018	–0.004	0.003	0.001	–0.006	0.008	0.759
Leaning Biden, *Concordant*	–0.043	–0.061	–0.026	0.001	0.008	–0.010	0.024	0.334
Leaning Biden, *Neutral*	–0.017	–0.032	–0.001	0.033	–0.003	–0.017	0.011	0.721
Leaning Biden, *Discordant*	–0.003	–0.015	0.009	0.628	0.007	–0.012	0.025	0.459
Leaning Trump, *Concordant*	0.007	–0.012	0.028	0.532	–0.002	–0.021	0.018	0.876
Leaning Trump, *Neutral*	–0.004	–0.023	0.013	0.637	–0.009	–0.027	0.007	0.248
Leaning Trump, *Discordant*	–0.002	–0.018	0.012	0.780	0.007	–0.015	0.026	0.508
Leaning Trump [*ref.: Leaning Biden*]	–0.016	–0.021	–0.011	0.001	0.034	0.029	0.039	< 0.001
Concordant [*ref.: Neutral*]	–0.057	–0.064	–0.051	0.001	0.041	0.036	0.047	< 0.001
Leaning Biden	–0.086	–0.094	–0.078	0.001	0.003	–0.004	0.011	0.384
Leaning Trump	–0.027	–0.036	–0.017	0.001	0.082	0.072	0.091	< 0.001
Discordant [*ref.: Neutral*]	0.092	0.087	0.097	< 0.001	0.168	0.162	0.174	< 0.001
Leaning Biden	0.091	0.084	0.098	< 0.001	0.193	0.184	0.201	< 0.001
Leaning Trump	0.093	0.085	0.101	< 0.001	0.141	0.132	0.151	< 0.001

Table S7. Estimation results for a hierarchical linear regression model with three-way interaction terms predicting the trustworthiness of a fact-check (7-point Likert scale normalized to the interval [0, 1]). Random intercepts for posts and subjects are included. N = 24,003 observations across 1,347 participants.

**Dependent Variable: Trustworthiness**					
**Variable**	**Coef.**	**Std. Error**	**Lower CI**	**Upper CI**	**P-value**
**Condition**					
Misleading (Expert Flag) [*ref.*]	–	–	–	–	–
Misleading (Community Flag)	–0.010	0.025	–0.059	0.039	0.685
Misleading (Community Note)	0.074**	0.024	0.027	0.121	0.002
**Political Leaning (Subject)**					
Biden [*ref.*]	–	–	–	–	–
Trump	–0.080**	0.025	–0.129	–0.030	0.002
**Political Concordance (Post)**					
Neutral [*ref.*]	–	–	–	–	–
Concordant	–0.031	0.018	–0.067	0.004	0.083
Discordant	0.035	0.018	–0.001	0.070	0.054
**Interactions**					
Concordant × Trump	0.035	0.020	–0.004	0.074	0.081
Discordant × Trump	–0.013	0.020	–0.052	0.026	0.527
Misleading (Community Flag) × Concordant	0.001	0.011	–0.021	0.024	0.897
Misleading (Community Note) × Concordant	–0.010	0.011	–0.031	0.012	0.373
Misleading (Community Flag) × Discordant	–0.001	0.011	–0.023	0.021	0.955
Misleading (Community Note) × Discordant	–0.029**	0.011	–0.051	–0.008	0.007
Misleading (Community Flag) × Trump	0.003	0.036	–0.068	0.074	0.940
Misleading (Community Note) × Trump	–0.032	0.035	–0.100	0.037	0.366
Misleading (Community Flag) × Concordant × Trump	–0.021	0.016	–0.053	0.011	0.201
Misleading (Community Note) × Concordant × Trump	–0.010	0.016	–0.041	0.022	0.543
Misleading (Community Flag) × Discordant × Trump	–0.006	0.016	–0.038	0.026	0.725
Misleading (Community Note) × Discordant × Trump	0.027	0.016	–0.004	0.059	0.086
Intercept	0.619***	0.021	0.579	0.659	<0.001
Subject-level RE	YES				
Post-level RE	YES				
AIC	–2,710.56				
Participants (n)	1,347				
Observations (N)	24,003				

Significance: $^{***}p < 0.001$; $^{**}p < 0.01$; $^{*}p < 0.05$

Table S8. Estimation results for hierarchical linear regression model with four-way interaction terms predicting the perceived misleadingness of a post (7-point Likert scale normalized to the interval [0, 1]). Random intercepts for posts and subjects are included. $N = $ 64,454 observations across 1,810 participants.

**Dependent Variable: Misleadingness**					
**Variable**	**Coef.**	**Std. Error**	**Lower CI**	**Upper CI**	**P-value**
**Condition**					
No Fact-Check [*ref.*]	–	–	–	–	–
Expert Flag	0.101***	0.010	0.080	0.121	<0.001
Community Flag	0.084***	0.011	0.063	0.105	<0.001
Community Note	0.146***	0.010	0.125	0.166	<0.001
**Political Leaning (Subject)**					
Biden [*ref.*]	–	–	–	–	–
Trump	–0.017	0.011	–0.038	0.003	0.101
**Political Concordance (Post)**					
Neutral [*ref.*]	–	–	–	–	–
Concordant	–0.077	0.050	–0.175	0.021	0.122
Discordant	0.138**	0.050	0.040	0.237	0.006
**Misleading (Post)**					
Misleading [*ref.*]	–	–	–	–	–
Non-misleading	–0.363***	0.050	–0.461	–0.265	<0.001
**Interactions**					
Concordant × Trump	0.066	0.051	–0.034	0.166	0.195
Discordant × Trump	–0.010	0.051	–0.110	0.089	0.836
Expert Flag × Concordant	0.004	0.012	–0.020	0.028	0.735
Community Flag × Concordant	–0.022	0.012	–0.047	0.002	0.072
Community Note × Concordant	–0.017	0.012	–0.041	0.006	0.152
Expert Flag × Discordant	–0.052***	0.013	–0.077	–0.028	<0.001
Community Flag × Discordant	–0.039**	0.013	–0.064	–0.014	0.003
Community Note × Discordant	–0.092***	0.012	–0.117	–0.068	<0.001
Expert Flag × Trump	–0.028	0.015	–0.058	0.001	0.059
Community Flag × Trump	–0.016	0.015	–0.046	0.014	0.293
Community Note × Trump	–0.029*	0.015	–0.058	0.000	0.046
Concordant × Non-misleading	0.089	0.071	–0.049	0.228	0.206
Discordant × Non-misleading	0.073	0.071	–0.066	0.212	0.304
Trump × Non-misleading	0.029*	0.012	0.005	0.053	0.018
Expert Flag × Non-misleading	–0.143***	0.012	–0.167	–0.119	<0.001
Community Flag × Non-misleading	–0.129***	0.012	–0.154	–0.105	<0.001
Community Note × Non-misleading	–0.172***	0.012	–0.195	–0.148	<0.001
Expert Flag × Concordant × Trump	–0.029	0.018	–0.064	0.006	0.110
Community Flag × Concordant × Trump	0.010	0.018	–0.026	0.045	0.598
Community Note × Concordant × Trump	–0.006	0.017	–0.041	0.028	0.717
Expert Flag × Discordant × Trump	0.025	0.018	–0.010	0.060	0.159
Community Flag × Discordant × Trump	0.013	0.018	–0.022	0.048	0.468
Community Note × Discordant × Trump	0.009	0.017	–0.025	0.043	0.605
Concordant × Trump × Non-misleading	0.023	0.072	–0.118	0.164	0.747
Discordant × Trump × Non-misleading	–0.035	0.072	–0.176	0.105	0.622
Expert Flag × Concordant × Non-misleading	–0.018	0.017	–0.052	0.016	0.302
Community Flag × Concordant × Non-misleading	0.019	0.018	–0.015	0.054	0.277
Community Note × Concordant × Non-misleading	0.001	0.017	–0.032	0.035	0.950
Expert Flag × Discordant × Non-misleading	0.021	0.017	–0.013	0.056	0.219
Community Flag × Discordant × Non-misleading	0.017	0.018	–0.018	0.052	0.348
Community Note × Discordant × Non-misleading	0.071***	0.017	0.037	0.106	<0.001
Expert Flag × Trump × Non-misleading	0.048**	0.018	0.014	0.082	0.006
Community Flag × Trump × Non-misleading	0.029	0.018	–0.006	0.064	0.100
Community Note × Trump × Non-misleading	0.052**	0.017	0.018	0.085	0.003
Expert Flag × Concordant × Trump × Non-misleading	0.007	0.025	–0.042	0.056	0.772
Community Flag × Concordant × Trump × Non-misleading	–0.033	0.025	–0.083	0.016	0.187

C. Additional analyses

C.1. Sharing intentions. *Note: Participants in our survey also had to state whether they would consider sharing the presented posts on social media. However, research shows that asking about misleadingness before assessing sharing intentions can influence the outcome variable, that is, increase correlation between the identification of misleadingness and sharing intentions (6). The following results should therefore be interpreted with caution.*

In our survey, participants assigned to the control condition had significantly lower (t-test: $t = -36.11$; $df = 13395$; $P < 0.001$, two-tailed) sharing intentions (7-point Likert scale normalized to the interval [0, 1]) for misleading ($M = 0.13$) than for non-misleading posts ($M = 0.23$). To quantify intervention effects, we fitted a hierarchical linear regression model with four-way interaction terms and random intercepts for subjects and posts to predict sharing intentions (see Section Methods of the main paper). Figure S3 shows the average marginal effects (see SI, Table S9 for AMEs).

All fact-checking interventions significantly reduced sharing intentions for misleading posts (see Figure S3a,c,d). Compared to the control condition, sharing intentions for misleading posts were, on average, 2.7 percentage points lower for participants exposed to expert flags (AME $= -0.027$; 95 % CI $= [-0.033, -0.021]$; $P = 0.001$), 3.5 percentage points lower for community flags (AME $= -0.035$; 95 % CI $= [-0.041, -0.029]$; $P = 0.001$), and 1.3 percentage points lower for community notes (AME $= -0.013$; 95 % CI $= [-0.018, -0.007]$; $P = 0.001$). In terms of percentages, these numbers translate to a reduction in sharing intentions of 21.3 % for expert flags, 27.6 % for community flags, and 10.3 % for community notes. Hence, compared to expert flags, community flags decreased sharing intentions for misleading posts (AME $= -0.008$; 95 % CI $= [-0.014, -0.003]$; $P = 0.001$), while community notes led to an increase (AME $= 0.014$; 95 % CI $= [0.014, 0.019]$; $P < 0.001$). The efficacy of the fact-checking interventions in reducing sharing intentions for misleading posts did not vary significantly for participants leaning towards Biden vs. Trump ($P = 0.817$ for expert flags, $P = 0.671$ for community flags, $P = 0.486$ for community notes). We further observe that Trump supporters had significantly higher baseline intentions to share misleading posts than Biden supporters (AME $= 0.018$; 95 % CI $= [0.014, 0.022]$; $P < 0.001$). Also, sharing intentions for misleading posts were, on average, higher for politically concordant posts (AME $= 0.022$; 95 % CI $= [0.017, 0.027]$; $P < 0.001$) than for politically discordant posts (AME $= -0.033$; 95 % CI $= [-0.038, -0.029]$; $P = 0.001$).

Similar to the previous analyses, the efficacy of community notes in reducing sharing intentions for misleading posts varied across political leanings (see Figure S3c). For Trump supporters, replacing an expert flag with a community note would have decreased sharing intentions for misleading posts by 2.2 percentage points if they were politically concordant (AME $= -0.022$; 95 % CI $= [-0.037, -0.008]$; $P = 0.001$). This effect was slightly positive for politically neutral (AME $= 0.015$; 95 % CI $= [0.002, 0.029]$; $P = 0.029$) and discordant posts (AME $= 0.035$; 95 % CI $= [0.022, 0.047]$; $P < 0.001$). We find a similar pattern for Biden supporters. Here, replacing an expert flag with a community note would have increased sharing intentions by 1.4 percentage points for politically discordant posts (AME $= 0.031$; 95 % CI $= [0.021, 0.042]$; $P < 0.001$), whereas the effects were smaller for politically neutral (AME $= 0.014$; 95 % CI $= [0.002, 0.026]$; $P = 0.018$) and not statistically significant for politically concordant posts (AME $= 0.010$; 95 % CI $= [-0.002, 0.023]$; $P = 0.100$).

We also observe some treatment condition effects on untagged non-misleading posts (see Figure S3b). For Biden supporters, displaying fact-checks on misleading posts increased sharing intentions for untagged non-misleading posts by 1.4 percentage points for expert flags (AME $= 0.012$; 95 % CI $= [0.006, 0.023]$; $P = 0.003$), by 2.7 percentage points for community flags (AME $= 0.027$; 95 % CI $= [0.018, 0.036]$; $P < 0.001$), and by 1.0 percentage points for community notes (AME $= 0.010$; 95 % CI $= [0.002, 0.019]$; $P = 0.020$). For Trump supporters, we do not observe significant effects across any of the conditions ($P = 0.081$ for expert flags, $P = 0.447$ for community flags, $P = 0.218$ for community notes). When comparing the average marginal effects across all participants (i.e., both Biden and Trump supporters), the treatment condition effects on untagged non-misleading posts were statistically significant for community flags (AME $= 0.012$; 95 % CI $= [0.005, 0.009]$; $P < 0.001$), but not for expert flags (AME $= 0.003$; 95 % CI $= [-0.004, 0.009]$; $P = 0.340$) and community notes (AME $= 0.002$; 95 % CI $= [-0.004, 0.008]$; $P = 0.511$). Furthermore, we find that Trump supporters had slightly higher baseline intentions to share non-misleading posts than Biden supporters (AME $= 0.006$; 95 % CI $= [0.002, 0.011]$; $P = 0.005$), and sharing intentions for non-misleading posts were higher for politically concordant posts (AME $= 0.028$; 95 % CI $= [0.021, 0.033]$; $P < 0.001$) than for politically discordant posts (AME $= -0.072$; 95 % CI $= [-0.078, -0.067]$; $P = 0.001$).

In sum, these results suggest that community notes were less successful in reducing sharing intentions for misleading posts than simple misinformation flags. In particular, politically discordant misleading posts with community notes were more likely to be shared (by both Biden and Trump supporters) relative to discordant posts with simple misinformation flags. A potential explanation might be that the community notes themselves were actually perceived as politically concordant (because they were applied to politically discordant original posts). Thus, participants may have been inclined to share the post because they were sharing the community note appended to it, rather than endorsing the original post.

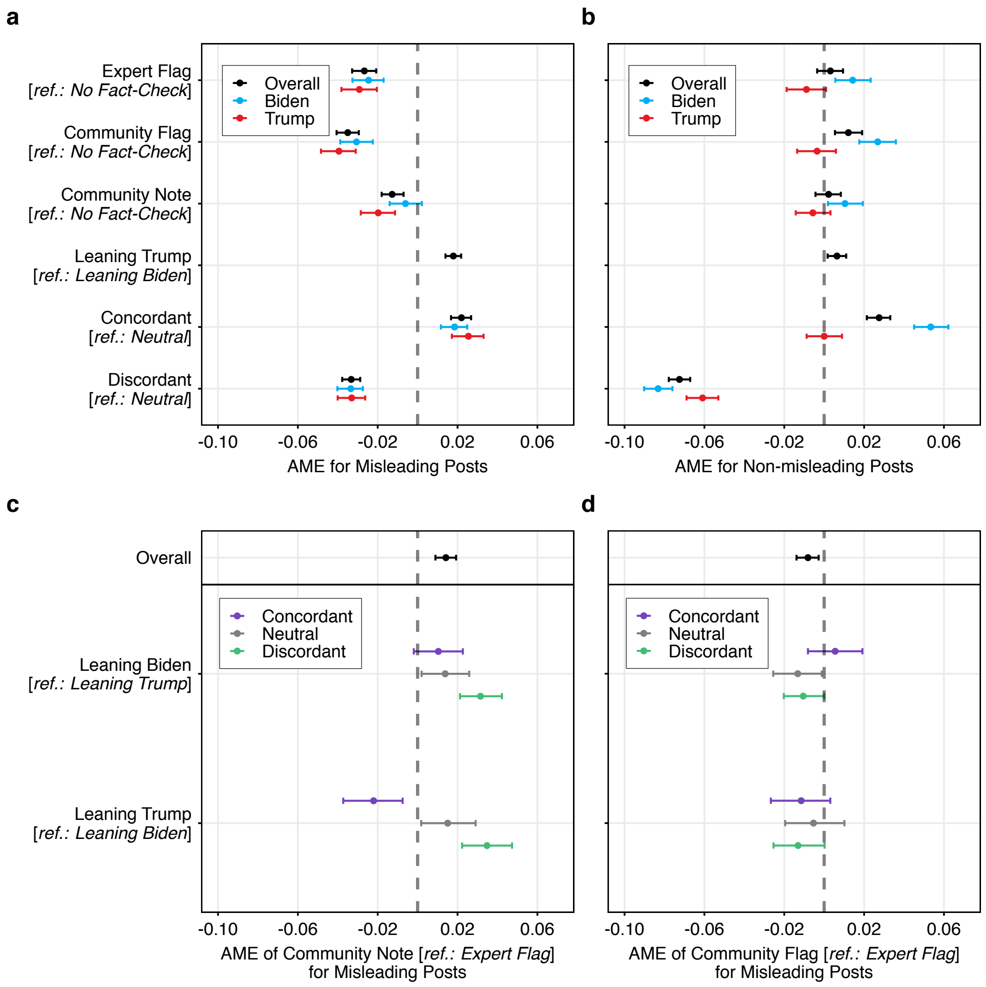

Fig. S3. Community notes did not consistently reduce sharing intentions for misleading posts. Shown are the average marginal effects (AME) and 95 % confidence intervals from a hierarchical linear regression model with interaction terms predicting sharing intentions (7-point Likert scale normalized to the interval [0, 1]). AME is the difference in the average predicted sharing intentions between the group of interest and the reference group (e.g., an AME of $+0.05$ indicates a 5 percentage point difference in sharing intentions). **(a)** AMEs for misleading posts. **(b)** AMEs for non-misleading posts. **(c)** AMEs when replacing an expert flag with a community note (i.e., the average difference between the predicted marginal effects for community notes vs. expert flags) across the political leanings of participants (leaning Trump vs. Biden) and the political congruence of the fact-checked posts (concordant, neutral, discordant). **(d)** AMEs when replacing an expert flag with a community flag (i.e., the average difference between the predicted marginal effects for community flags vs. expert flags) across the political leanings of participants and the fact-checked posts. Control variables and random intercepts for posts and subjects were included. The 95 % confidence intervals (error bars) were derived using the bootstrap method for 1,000 resamples. $N = 64{,}454$ observations across 1,810 participants. Full estimation results are in SI, Table S9.

Table S9. Average marginal effects (AME) and 95 % confidence intervals from a hierarchical linear regression model with four-way interaction terms predicting sharing intentions (7-point Likert scale normalized to the interval [0, 1]). AME is the difference in average predicted sharing intentions between the group of interest and the reference group (e.g., an AME of $+0.05$ indicates a 5 percentage point difference in sharing intentions). AMEs are reported separately for misleading (columns 2–5) and non-misleading posts (columns 6–9). The indented rows report the AMEs of an intervention depending on the political leanings of participants (leaning Trump vs. Biden) and the political congruence of the fact-checked posts (concordant, neutral, discordant). Random intercepts for posts and subjects are included. The 95 % confidence intervals for the AMEs were derived using the bootstrap method for 1,000 resamples. $N =$ 64,454 observations across 1,810 participants.

	**Misleading**				**Non-misleading**			
	**AME**	**Lower CI**	**Upper CI**	**P-value**	**AME**	**Lower CI**	**Upper CI**	**P-value**
Expert Flag [*ref.: No Fact-Check*]	–0.027	–0.033	–0.021	0.001	0.003	–0.004	0.009	0.340
Leaning Biden	–0.025	–0.033	–0.017	0.001	0.014	0.006	0.023	0.003
Leaning Trump	–0.029	–0.038	–0.020	0.001	–0.009	–0.019	0.001	0.081
Community Flag [*ref.: No Fact-Check*]	–0.035	–0.041	–0.029	0.001	0.012	0.005	0.019	< 0.001
Leaning Biden	–0.031	–0.039	–0.022	0.001	0.027	0.018	0.036	< 0.001
Leaning Trump	–0.039	–0.048	–0.031	0.001	–0.004	–0.014	0.006	0.447
Community Note [*ref.: No Fact-Check*]	–0.013	–0.018	–0.007	0.001	0.002	–0.004	0.008	0.511
Leaning Biden	–0.006	–0.014	0.002	0.135	0.010	0.002	0.019	0.020
Leaning Trump	–0.020	–0.028	–0.011	0.001	–0.006	–0.014	0.003	0.218
Community Note [*ref.: Expert Flag*]	0.014	0.009	0.019	< 0.001	–0.001	–0.007	0.005	0.789
Leaning Biden, *Concordant*	0.010	–0.002	0.023	0.100	–0.011	–0.027	0.006	0.188
Leaning Biden, *Neutral*	0.014	0.002	0.026	0.018	0.006	–0.010	0.021	0.441
Leaning Biden, *Discordant*	0.031	0.021	0.042	< 0.001	–0.007	–0.019	0.006	0.276
Leaning Trump, *Concordant*	–0.022	–0.037	–0.008	0.001	0.001	–0.017	0.018	0.858
Leaning Trump, *Neutral*	0.015	0.002	0.029	0.029	0.002	–0.015	0.019	0.833
Leaning Trump, *Discordant*	0.035	0.022	0.047	< 0.001	0.006	–0.010	0.021	0.417
Community Flag [*ref.: Expert Flag*]	–0.008	–0.014	–0.003	0.001	0.009	0.002	0.016	0.010
Leaning Biden, *Concordant*	0.006	–0.008	0.019	0.442	0.023	0.006	0.041	0.010
Leaning Biden, *Neutral*	–0.013	–0.026	–0.001	0.037	0.023	0.008	0.039	0.004
Leaning Biden, *Discordant*	–0.010	–0.020	<0.001	0.053	–0.008	–0.021	0.005	0.220
Leaning Trump, *Concordant*	–0.012	–0.027	0.003	0.140	0.013	–0.005	0.033	0.150
Leaning Trump, *Neutral*	–0.005	–0.020	0.010	0.445	0.007	–0.009	0.026	0.391
Leaning Trump, *Discordant*	–0.013	–0.025	<0.001	0.053	–0.004	–0.019	0.011	0.598
Leaning Trump [*ref.: Leaning Biden*]	0.018	0.014	0.022	< 0.001	0.006	0.002	0.011	0.005
Concordant [*ref.: Neutral*]	0.022	0.017	0.027	< 0.001	0.028	0.021	0.033	< 0.001
Leaning Biden	0.018	0.012	0.025	< 0.001	0.053	0.045	0.062	< 0.001
Leaning Trump	0.025	0.017	0.033	< 0.001	<0.001	–0.009	0.009	0.986
Discordant [*ref.: Neutral*]	–0.033	–0.038	–0.029	0.001	–0.072	–0.078	–0.067	0.001
Leaning Biden	–0.033	–0.040	–0.027	0.001	–0.083	–0.090	–0.076	0.001
Leaning Trump	–0.033	–0.040	–0.026	0.001	–0.061	–0.069	–0.053	0.001

C.2. Demographics and beliefs. We repeated our regression analyses including information on the participants' demographics and beliefs (see Section Demographics, beliefs, and cognitive reflection in the main paper). This included the participants' gender (male, female, non-binary), level of education (attended college vs. didn't attend college), stance towards God (believes in God vs. doesn't believe in God), ethnicity (ethnic minority vs. not a minority), and COVID-19 vaccination status (vaccinated vs. not vaccinated). In addition, we included the following variables regarding the participants attitude: willingness to take risks (low vs.

high), trust in people they interact with in their daily life (low vs. high), trust in democracy (low vs. high), and preference to perform tasks that require thinking (low thinking preference vs. high thinking preference). For all Likert-scale variables, a value of greater than "undecided" (3) was considered high (otherwise low).

Table S10 reports the AMEs for misleadingness ratings under the control condition. We further studied moderation effects, i.e., how the efficacy of fact-checking interventions varied depending on demographics and beliefs. The corresponding AMEs are reported in Tables S11 to S13.

Table S10. AMEs of demographics and beliefs on perceived misleadingness under the control condition (No Fact-Check) for misleading posts. The AMEs are calculated from hierarchical linear regression models with interaction terms. Random intercepts for posts and subjects are included. The 95 % confidence intervals for the AMEs were derived using the bootstrap method for 1,000 resamples. $N =$ **64,454 observations across 1,810 participants for DV:** $Misleadingness$

	Misleading			
	**AME**	**Lower CI**	**Upper CI**	P**-value**
**Dependent Variable: Misleadingness**				
Female [*ref.: Male*]	0.000	–0.012	0.012	0.970
Attended college [*ref.: Didn't attend college*]	–0.003	–0.014	0.009	0.639
Age: $<$ 30 years [*ref.: 30 – 50 years*]	0.002	–0.013	0.017	0.760
Age: $>$ 50 years [*ref.: 30 – 50 years*]	0.002	–0.010	0.017	0.777
Believes in God [*ref.: Doesn't believe in God*]	–0.005	–0.019	0.007	0.398
Minority [*ref.: Not a minority*]	–0.020	–0.032	–0.007	0.007
Vaccinated against COVID-19 [*ref.: Not vaccinated against COVID-19*]	0.023	0.009	0.037	0.004
Low willingness to take risks [*ref.: High willingness to take risks*]	–0.025	–0.037	–0.013	0.001
High trust [*ref.: Low Trust*]	0.002	–0.010	0.014	0.796
High trust in democracy [*ref.: Low trust in democracy*]	0.008	–0.003	0.020	0.149
High thinking preference [*ref.: Low thinking preference*]	0.006	–0.006	0.018	0.323

Table S11. AME of replacing an expert flag with a community note on trust in fact-checks for misleading posts depending on participants' demographics and beliefs. The AMEs are calculated from a hierarchical linear regression model with interaction terms predicting predicting the trustworthiness of a fact-check (7-point Likert scale normalized to the interval [0, 1]). Random intercepts for posts and subjects are included. The 95 % confidence intervals for the AMEs were derived using the bootstrap method for 1,000 resamples. N = 24,003 observations across 1,347 participants.

Dependent Variable: Trustworthiness				
	**Misleading**			
	**AME**	**Lower CI**	**Upper CI**	**P-value**
**AMEs of Community Note *[ref. Expert Flag]***				
Female	0.036	0.026	0.045	< 0.001
Male	0.053	0.044	0.062	< 0.001
Didn't attend college	0.041	0.033	0.050	< 0.001
Attended college	0.050	0.041	0.060	< 0.001
Age: $<$ 30 years	0.054	0.041	0.067	< 0.001
Age: 30–49 years	0.046	0.037	0.055	< 0.001
Age: $>$ 50 years	0.038	0.027	0.049	< 0.001
Doesn't believe in God	0.077	0.068	0.086	< 0.001
Believes in God	0.017	0.007	0.026	< 0.001
Not a Minority	0.038	0.031	0.046	< 0.001
Minority	0.067	0.053	0.078	< 0.001
Vaccinated against COVID-19	0.045	0.037	0.052	< 0.001
Not vaccinated against COVID-19	0.046	0.033	0.058	< 0.001
Low willingness to take risks	0.030	0.020	0.040	< 0.001
High willingness to take risks	0.056	0.048	0.065	< 0.001
Low trust	0.035	0.023	0.047	< 0.001
High trust	0.049	0.042	0.058	< 0.001
Low trust in democracy	0.031	0.022	0.040	< 0.001
High trust in democracy	0.060	0.051	0.068	< 0.001
Low thinking preference	0.049	0.041	0.057	< 0.001
High thinking preference	0.040	0.030	0.050	< 0.001

Table S12. AME of replacing an expert flag with a community note on misleadingness ratings for misleading posts depending on participants' demographics and beliefs. The AMEs are calculated from a hierarchical linear regression model with interaction terms predicting the perceived misleadingness of a post (7-point Likert scale normalized to the interval [0, 1]). Random intercepts for posts and subjects are included. The 95 % confidence intervals for the AMEs were derived using the bootstrap method for 1,000 resamples. $N =$ 64,454 observations across 1,810 participants.

Dependent Variable: Misleadingness				
	Misleading			
	AME	Lower CI	Upper CI	P-value
**AMEs of Community Note *[ref. Expert Flag]***				
Female	0.035	0.026	0.044	< 0.001
Male	0.008	–0.002	0.017	0.118
Didn't attend college	0.027	0.019	0.036	< 0.001
Attended college	0.017	0.007	0.028	< 0.001
Age: $<$ 30 years	0.030	0.018	0.044	< 0.001
Age: 30–49 years	0.017	0.007	0.027	< 0.001
Age: $>$ 50 years	0.027	0.015	0.039	< 0.001
Doesn't believe in God	0.030	0.022	0.038	< 0.001
Believes in God	0.017	0.007	0.026	0.003
Not a Minority	0.023	0.015	0.030	< 0.001
Minority	0.023	0.010	0.037	< 0.001
Vaccinated against COVID-19	0.017	0.010	0.025	< 0.001
Not vaccinated against COVID-19	0.039	0.025	0.051	< 0.001
Low willingness to take risks	0.001	–0.009	0.011	0.831
High willingness to take risks	0.039	0.031	0.047	< 0.001
Low trust	0.034	0.022	0.046	< 0.001
High trust	0.018	0.010	0.026	< 0.001
Low trust in democracy	0.031	0.022	0.041	< 0.001
High trust in democracy	0.014	0.005	0.023	0.003
Low thinking preference	0.025	0.016	0.033	< 0.001
High thinking preference	0.020	0.010	0.030	< 0.001

Table S13. AME of different fact-checking interventions (reference: control condition) on the identification of misleading posts depending on participants' demographics and beliefs. The AMEs are calculated from a hierarchical linear regression model with interaction terms predicting misleadingness (7-point Likert scale normalized to the interval [0, 1]). The 95 % confidence intervals for the AMEs were derived using the bootstrap method for 1,000 resamples. $N =$ 64,454 observations across 1,810 participants.

Dependent Variable: Misleadingness				
	Misleading			
	AME	Lower CI	Upper CI	P-value
**AMEs of Expert Flag** ***[ref. No Fact-Check]***				
Female	0.057	0.047	0.067	$<$ 0.001
Male	0.086	0.076	0.097	$<$ 0.001
Didn't attend college	0.069	0.059	0.079	$<$ 0.001
Attended college	0.073	0.062	0.084	$<$ 0.001
Age: $<$ 30 years	0.072	0.056	0.087	$<$ 0.001
Age: 30–49 years	0.072	0.062	0.083	$<$ 0.001
Age: $>$ 50 years	0.068	0.054	0.081	$<$ 0.001
Doesn't believe in God	0.074	0.065	0.084	$<$ 0.001
Believes in God	0.068	0.057	0.078	$<$ 0.001
Not a Minority	0.071	0.063	0.080	$<$ 0.001
Minority	0.069	0.056	0.083	$<$ 0.001
Vaccinated against COVID-19	0.076	0.068	0.085	$<$ 0.001
Not vaccinated against COVID-19	0.055	0.039	0.070	$<$ 0.001
Low willingness to take risks	0.087	0.077	0.099	$<$ 0.001
High willingness to take risks	0.058	0.049	0.069	$<$ 0.001
Low trust	0.055	0.041	0.069	$<$ 0.001
High trust	0.078	0.069	0.086	$<$ 0.001
Low trust in democracy	0.059	0.049	0.069	$<$ 0.001
High trust in democracy	0.083	0.072	0.093	$<$ 0.001
Low thinking preference	0.075	0.066	0.084	$<$ 0.001
High thinking preference	0.064	0.053	0.075	$<$ 0.001
**AMEs of Community Flag** ***[ref. No Fact-Check]***				
Female	0.048	0.039	0.058	$<$ 0.001
Male	0.073	0.062	0.084	$<$ 0.001
Didn't attend college	0.056	0.046	0.065	$<$ 0.001
Attended college	0.064	0.053	0.076	$<$ 0.001
Age: $<$ 30 years	0.058	0.043	0.074	$<$ 0.001
Age: 30–49 years	0.059	0.048	0.069	$<$ 0.001
Age: $>$ 50 years	0.061	0.047	0.075	$<$ 0.001
Doesn't believe in God	0.061	0.051	0.071	$<$ 0.001
Believes in God	0.058	0.048	0.068	$<$ 0.001
Not a Minority	0.055	0.046	0.064	$<$ 0.001
Minority	0.074	0.059	0.088	$<$ 0.001
Vaccinated against COVID-19	0.061	0.053	0.070	$<$ 0.001
Not vaccinated against COVID-19	0.055	0.039	0.072	$<$ 0.001
Low willingness to take risks	0.076	0.064	0.087	$<$ 0.001
High willingness to take risks	0.047	0.038	0.057	$<$ 0.001
Low trust	0.051	0.037	0.066	$<$ 0.001
High trust	0.063	0.054	0.071	$<$ 0.001
Low trust in democracy	0.053	0.043	0.063	$<$ 0.001
High trust in democracy	0.066	0.056	0.077	$<$ 0.001
Low thinking preference	0.065	0.056	0.074	$<$ 0.001
High thinking preference	0.051	0.039	0.063	$<$ 0.001
**AMEs of Community Note** ***[ref. No Fact-Check]***				
Female	0.092	0.082	0.101	$<$ 0.001
Male	0.094	0.084	0.105	$<$ 0.001
Didn't attend college	0.096	0.087	0.105	$<$ 0.001
Attended college	0.090	0.080	0.100	$<$ 0.001
Age: $<$ 30 years	0.102	0.087	0.116	$<$ 0.001
Age: 30–49 years	0.089	0.079	0.099	$<$ 0.001

C.3. Reliance on fact-checks. Participants in the treatment conditions were also asked to indicate whether they tend to rely on fact-checks in general (None (1) to Extreme (5)). We repeated our analysis with reliance on fact-checks as an additional explanatory variable (see Section Demographics, beliefs, and cognitive reflection in the main paper). Here, we code responses higher than "Moderate" (3) as a high reliance in fact-checks (otherwise = low). Table S14 reports the AMEs, i.e., how the efficacy of fact-checking interventions varied depending on participants' self-reported reliance on fact-checks across our three dependent variables.

Table S14. Average marginal effects (AME) of replacing an expert flag with a community note depending on participants' reliance on fact-checks. The AME are calculated from hierarchical linear regression models with interaction terms predicting trust in fact-checks and misleadingness for misleading posts. Random intercepts for posts and subjects are included. The 95 % confidence intervals for the AMEs were derived using the bootstrap method for 1,000 resamples. $N = $ 24,003 observations across 1,347 participants for DV: $Trustworthiness$, and $N = $ 48,249 observations across 1,347 participants for DV: $Misleadingness$

	**Misleading**			
	**AME**	**Lower CI**	**Upper CI**	**P-value**
**Dependent Variable: Trustworthiness**				
**AMEs of Community Note *[ref. Expert Flag]***				
High reliance on fact-checks	0.038	0.030	0.045	< 0.001
Low reliance on fact-checks	0.005	–0.008	0.017	0.439
**Dependent Variable: Misleadingness**				
**AMEs of Community Note *[ref. Expert Flag]***				
High reliance on fact-checks	0.012	0.004	0.019	0.005
Low reliance on fact-checks	0.035	0.023	0.047	< 0.001

C.4. Cognitive Reflection Test (CRT). A common method to assess the level of a persons' reflective thinking is the so-called Cognitive Reflection Test (CRT). Participants in our study were asked to answer a 4-item CRT (for further details see Section D). We classified four correct answers as "Passed CRT" and less than four correct answers as "Failed CRT". Table S15 reports the average marginal effects (AME) across our three dependent variables. Moderation effects, i.e., how the efficacy of fact-checking interventions varied depending on the outcomes of the CRT are reported in Table S16. The findings are described in detail in Section Demographics, beliefs, and cognitive reflection of the main paper.

Table S15. Average marginal effects (AME) of the outcome of a CRT ($= 1$ if passed; $= 0$ otherwise) on perceived misleadingness for misleading posts. The AME are calculated from linear mixed-effects regression models with interaction terms. Random intercepts for posts and subjects are included. The 95 % confidence intervals for the AMEs were derived using the bootstrap method for 1,000 resamples. $N =$ 64,454 observations across 1,810 participants for DV: $Misleadingness$

	Misleading			
	**AME**	**Lower CI**	**Upper CI**	**P-value**
**Dependent Variable: Misleadingness**				
Passed CRT [*ref.: Failed CRT*]	0.034	0.023	0.045	< 0.001

Table S16. Average marginal effects (AME) of different fact-checking interventions depending on the outcome of the CRT ($= 1$ if passed; $= 0$ otherwise) for misleading posts. The AME are calculated from linear mixed-effects regression models with interaction terms predicting trust in fact-checks and perceived misleadingness. Random intercepts for posts and subjects are included. The 95 % confidence intervals for the AMEs were derived using the bootstrap method for 1,000 resamples. $N =$ 24,003 observations across 1,347 participants for DV: $Trustworthiness$, and $N =$ 64,454 observations across 1,810 participants for DV: $Misleadingness$.

	Misleading			
	**AME**	**Lower CI**	**Upper CI**	**P-value**
**Dependent Variable: Trustworthiness**				
**AMEs of Community Note *[ref. Expert Flag]***				
Passed CRT	0.074	0.065	0.083	< 0.001
Failed CRT	0.026	0.017	0.035	< 0.001
**Dependent Variable: Misleadingness**				
**AMEs of Community Note *[ref. Expert Flag]***				
Passed CRT	0.034	0.025	0.043	< 0.001
Failed CRT	0.017	0.009	0.026	< 0.001
**AMEs of Community Note *[ref. No Fact-Check]***				
Passed CRT	0.094	0.084	0.104	< 0.001
Failed CRT	0.094	0.084	0.103	< 0.001
**AMEs of Community Flag *[ref. No Fact-Check]***				
Passed CRT	0.048	0.038	0.059	< 0.001
Failed CRT	0.066	0.056	0.076	< 0.001
**AMEs of Expert Flag *[ref. No Fact-Check]***				
Passed CRT	0.060	0.048	0.071	< 0.001
Failed CRT	0.077	0.068	0.086	< 0.001

C.5. Analysis with hierarchical logistic regression models. We repeated our analysis with an alternative model specification using hierarchical logistic regression model and treating the Likert-scale responses as binary variables. Specifically, the dependent variable *Trustworthy* took the value $= 1$ if the fact-check was rated at least as somewhat trustworthy (i.e., participants gave a 5 or higher on the 7-point Likert scale) and $= 0$ otherwise. The dependent variable *Misleading* took the value $= 1$ if the fact-check was rated at least as somewhat misleading (i.e., participants gave a 5 or higher on the 7-point Likert scale) and $= 0$ otherwise. Table S17 reports the corresponding frequencies across the four experimental conditions. All explanatory variables and random effects specifications were the same as in our main analysis. The logistic mixed-effects models were implemented in R 4.3.2 using the `glmer` package and the `marginaleffects` package.

Consistent with our main analysis, we observe that users exposed to community notes perceived fact-checks as significantly more trustworthy (see Table S18) than those exposed to simple misinformation flags (all $P < 0.01$). Furthermore, all fact-checking interventions resulted in participants rating misleading posts as significantly more misleading (see Table S19). In sum, we find that all main findings are robust with similar effect sizes as in our main analysis.

Table S17. Frequency of ratings on the level of response items (per post and per participant) across the experimental conditions. Ratings given by the participants on a 7-point Likert scale were rescaled into binary variables that took the value $= 1$ if the rating was greater than Neutral (and $= 0$ otherwise).

Variable	Overall	No Fact-Check	Expert Flag	Community Flag	Community Note
Observations (N)	64,454	16,205	15,885	15,192	17,172
**Trustworthy**					
Yes	14,514 (60%)	–	4,617 (59%)	4,260 (56%)	5,637 (66%)
No	9,489 (40%)	–	3,204 (41%)	3,336 (44%)	2,949 (34%)
**Misleading**					
Yes	43,099 (67%)	10,755 (66%)	10,473 (66%)	9,998 (66%)	11,873 (69%)
No	21,355 (33%)	5,450 (34%)	5,412 (34%)	5,194 (34%)	5,299 (31%)

Table S18. Average marginal effects (AME) and 95 % confidence intervals from a hierarchical logistic regression model with three-way interaction terms predicting whether a fact-check was rated as trustworthy ($0 = no$; $1 = yes$). AME is the difference in average predicted probability of whether a fact-check was rated as trustworthy between the group of interest and the reference group expressed as a proportion (e.g., an AME of $+0.05$ indicates a 5 percentage point difference in predicted probabilities). The indented rows report the AMEs of an intervention depending on the political leanings of participants (leaning Trump vs. Biden) and the political congruence of the fact-checked posts (concordant, neutral, discordant). Random intercepts for posts and subjects are included. The 95 % confidence intervals for the AMEs were derived using the bootstrap method for 500 resamples. N = 24,003 observations across 1,347 participants.

	**AME**	**Lower CI**	**Upper CI**	**P-value**
Community Note [*ref.: Expert Flag*]	0.065	0.055	0.074	< 0.001
Leaning Biden, *Concordant*	0.104	0.079	0.129	< 0.001
Leaning Biden, *Neutral*	0.120	0.098	0.144	< 0.001
Leaning Biden, *Discordant*	0.047	0.025	0.068	< 0.001
Leaning Trump, *Concordant*	0.013	–0.014	0.041	0.360
Leaning Trump, *Neutral*	0.052	0.026	0.080	< 0.001
Leaning Trump, *Discordant*	0.047	0.023	0.073	< 0.001
Community Flag [*ref.: Expert Flag*]	–0.024	–0.034	–0.014	0.002
Leaning Biden, *Concordant*	–0.024	–0.057	0.007	0.140
Leaning Biden, *Neutral*	–0.013	–0.039	0.012	0.286
Leaning Biden, *Discordant*	–0.019	–0.041	0.002	0.084
Leaning Trump, *Concordant*	–0.047	–0.075	–0.019	0.006
Leaning Trump, *Neutral*	–0.024	–0.054	0.004	0.088
Leaning Trump, *Discordant*	–0.017	–0.045	0.009	0.194
Leaning Trump [*ref.: Leaning Biden*]	–0.100	–0.109	–0.092	0.002
Concordant [*ref.: Neutral*]	–0.043	–0.055	–0.032	0.002
Discordant [*ref.: Neutral*]	0.048	0.038	0.058	< 0.001

Table S19. Average marginal effects (AME) and 95 % confidence intervals from a hierarchical logistic regression model with four-way interaction terms predicting whether a post was rated as misleading ($0 = no$; $1 = yes$). AME is the difference in average predicted probability of whether a fact-check was rated as misleading between the group of interest and the reference group expressed as a proportion (e.g., an AME of $+0.05$ indicates a 5 percentage point difference in predicted probabilities). AMEs are reported separately for misleading (columns 2–5) and non-misleading posts (columns 6–9). The indented rows report the AMEs of an intervention depending on the political leanings of participants (leaning Trump vs. Biden) and the political congruence of the fact-checked posts (concordant, neutral, discordant). Random intercepts for posts and subjects are included. The 95 % confidence intervals for the AMEs were derived using the bootstrap method for 500 resamples. N = 64,454 observations across 1,810 participants.

	**Misleading**				**Non-misleading**			
	**AME**	**Lower CI**	**Upper CI**	**P-value**	**AME**	**Lower CI**	**Upper CI**	**P-value**
Expert Flag [*ref.: No Fact-Check*]	0.067	0.057	0.077	< 0.001	–0.078	–0.091	–0.064	0.002
Community Flag [*ref.: No Fact-Check*]	0.050	0.041	0.060	< 0.001	–0.072	–0.086	–0.060	0.002
Community Note [*ref.: No Fact-Check*]	0.080	0.071	0.090	< 0.001	–0.033	–0.046	–0.019	0.002
Community Note [*ref.: Expert Flag*]	0.013	0.006	0.021	0.006	0.046	0.033	0.060	< 0.001
Leaning Biden, *Concordant*	0.032	0.010	0.055	0.008	0.023	–0.005	0.052	0.088
Leaning Biden, *Neutral*	–0.002	–0.015	0.011	0.728	0.054	0.026	0.085	0.006
Leaning Biden, *Discordant*	–0.014	–0.024	–0.004	0.012	0.068	0.037	0.104	< 0.001
Leaning Trump, *Concordant*	0.068	0.045	0.094	< 0.001	0.080	0.046	0.112	< 0.001
Leaning Trump, *Neutral*	0.028	0.008	0.049	0.012	0.026	–0.006	0.060	0.122
Leaning Trump, *Discordant*	–0.032	–0.047	–0.016	0.002	0.023	–0.012	0.054	0.194
Community Flag [*ref.: Expert Flag*]	–0.017	–0.025	–0.008	0.002	0.006	–0.007	0.019	0.374
Leaning Biden, *Concordant*	–0.061	–0.086	–0.036	0.002	0.010	–0.020	0.039	0.496
Leaning Biden, *Neutral*	–0.009	–0.025	0.008	0.254	0.005	–0.026	0.037	0.742
Leaning Biden, *Discordant*	–0.009	–0.019	0.001	0.106	0.023	–0.011	0.059	0.196
Leaning Trump, *Concordant*	0.009	–0.018	0.039	0.566	0.022	–0.017	0.059	0.246
Leaning Trump, *Neutral*	–0.003	–0.027	0.019	0.798	–0.022	–0.055	0.011	0.204
Leaning Trump, *Discordant*	–0.023	–0.040	–0.007	0.010	–0.004	–0.037	0.033	0.838
Leaning Trump [*ref.: Leaning Biden*]	–0.029	–0.036	–0.022	0.002	0.049	0.039	0.058	< 0.001
Concordant [*ref.: Neutral*]	–0.070	–0.079	–0.061	0.002	0.042	0.029	0.053	< 0.001
Discordant [*ref.: Neutral*]	0.053	0.046	0.060	< 0.001	0.232	0.221	0.244	< 0.001

C.6. Analysis with cluster-robust standard errors. In our main analysis, we used hierarchical linear regression models with crossed random intercepts for posts and subjects. As an alternative model specification, we repeated our analysis using a linear regression model with robust standard errors clustered on both subjects and posts, analogous to earlier research (7). The average marginal effects (AMEs) are visualized in Figures S4 and S5 and tabulated in Tables S20 and S21. Across all models, the results were qualitatively identical and consistently supported our findings.

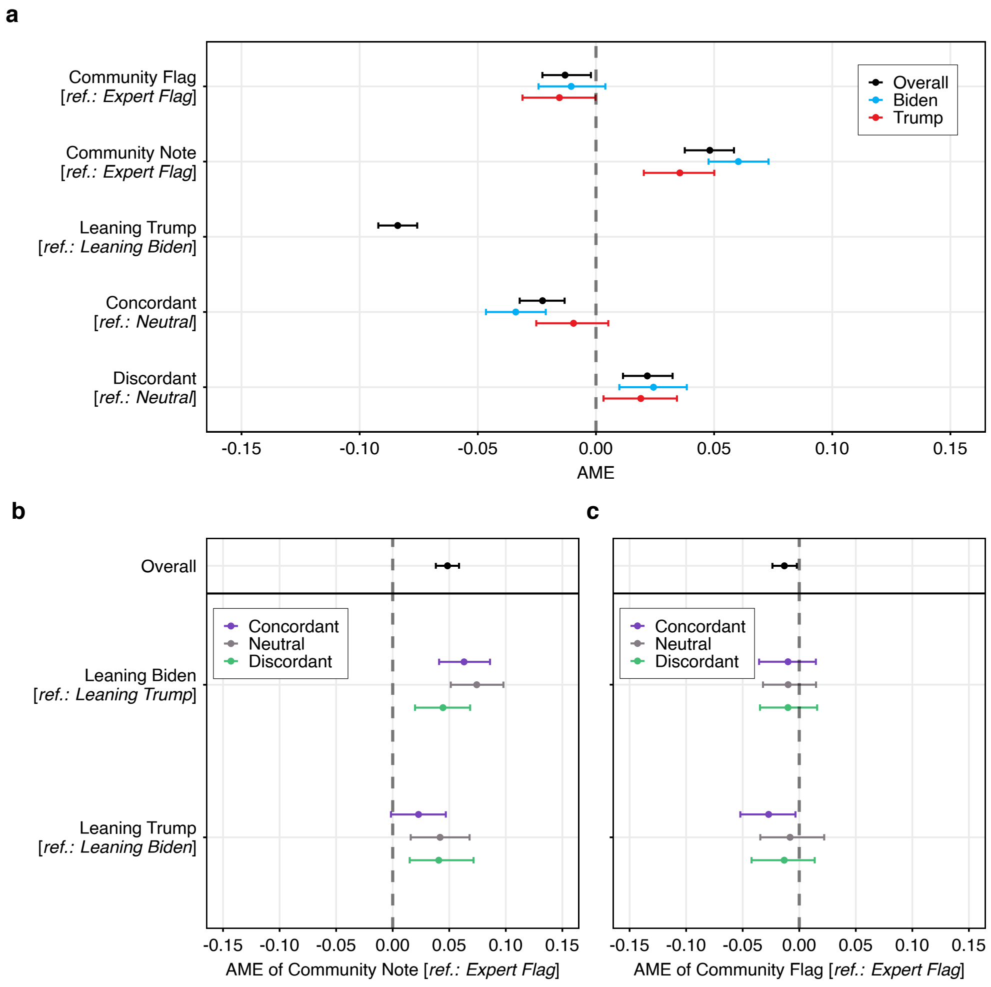

Fig. S4. **(a)** Shown are the average marginal effects (AME) and 95 % confidence intervals from a linear regression model with robust standard errors clustered on subjects and posts predicting the trustworthiness of a fact-check (7-point Likert scale normalized to the interval [0, 1]). **(b)** AME of replacing an expert flag with a community note (i.e., the average difference between the predicted marginal effects for community notes vs. expert flags) across the political leanings of participants (leaning Trump vs. Biden) and the political congruence of the fact-checked posts (concordant, neutral, discordant). **(c)** AME of replacing an expert flag with a community flag (i.e., the average difference between the predicted marginal effects for community flags vs. expert flags) across the political leanings of participants and the fact-checked posts. Control variables and random intercepts for posts and subjects were included. The 95 % confidence intervals (error bars) were derived using the bootstrap method for 1,000 resamples. $N = 24{,}003$ observations across 1,347 participants. Full AMEs are in SI, Table S20.

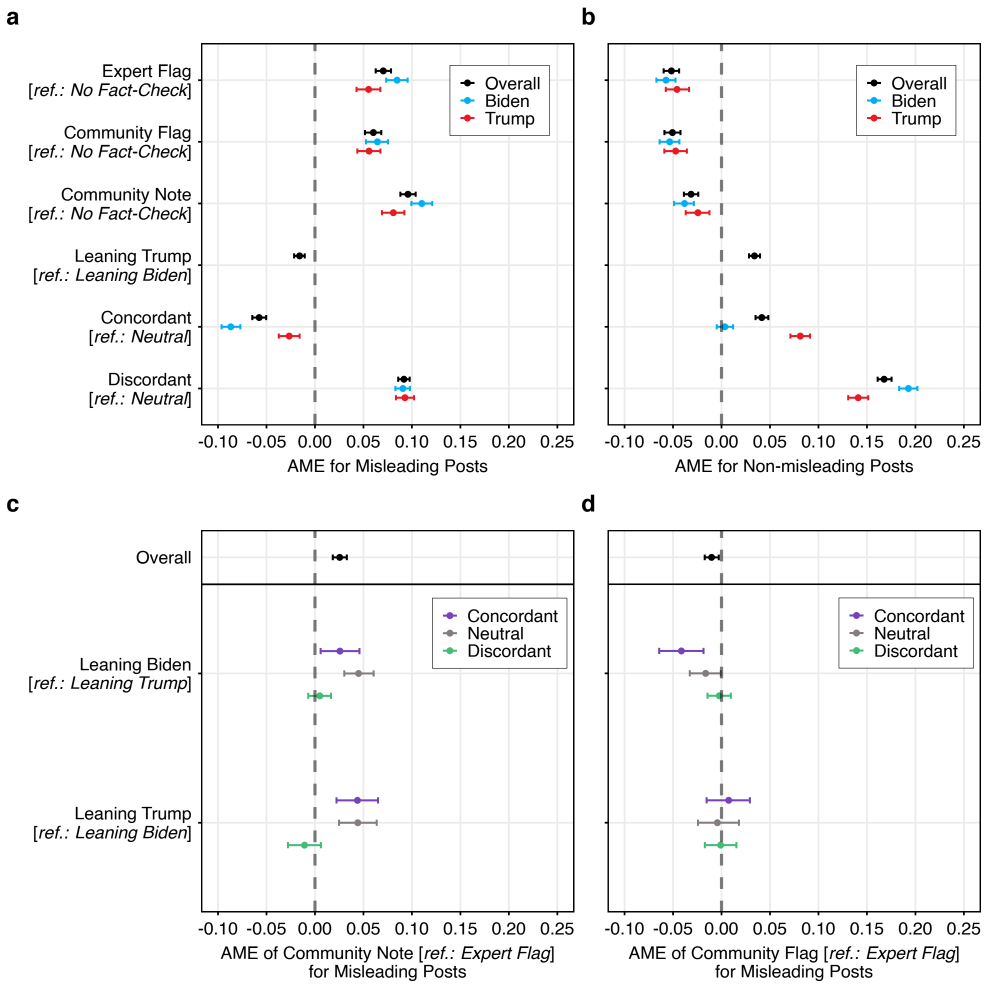

Fig. S5. Shown are the average marginal effects (AME) and 95 % confidence intervals from a linear regression model with robust standard errors clustered on subjects and posts predicting the perceived misleadingness of a post (7-point Likert scale normalized to the interval [0, 1]). **(a)** AMEs for misleading posts. **(b)** AMEs for non-misleading posts. **(c)** AMEs when replacing an expert flag with a community note (i.e., the average difference between the predicted marginal effects for community notes vs. expert flags) across the political leanings of participants (leaning Trump vs. Biden) and the political congruence of the fact-checked posts (concordant, neutral, discordant). **(d)** AMEs when replacing an expert flag with a community flag (i.e., the average difference between the predicted marginal effects for community flags vs. expert flags) across the political leanings of participants and the fact-checked posts. Control variables and random intercepts for posts and subjects were included. The 95 % confidence intervals (error bars) were derived using the bootstrap method for 1,000 resamples. $N = 64{,}454$ observations across 1,810 participants. Full AMEs are in SI, Table S21.

Table S20. Average marginal effects (AME) and 95 % confidence intervals from a linear regression model with robust standard errors clustered on subjects and posts predicting the trustworthiness of a fact-check (7-point Likert scale normalized to the interval [0, 1]). AME is the difference in average predicted probability of whether a fact-check was rated as trustworthy between the group of interest and the reference group expressed as a proportion (e.g., an AME of $+0.05$ indicates a 5 percentage point difference in predicted probabilities). The indented rows report the AMEs of an intervention depending on the political leanings of participants (leaning Trump vs. Biden) and the political congruence of the fact-checked posts (concordant, neutral, discordant). Random intercepts for posts and subjects are included. The 95 % confidence intervals for the AMEs were derived using the bootstrap method for 1,000 resamples. $N = 24{,}003$ observations across 1,347 participants.

	Misleading			
	**AME**	**Lower CI**	**Upper CI**	**P-value**
Community Note [*ref.: Expert Flag*]	0.048	0.038	0.059	< 0.001
Leaning Biden, *Concordant*	0.063	0.041	0.086	< 0.001
Leaning Biden, *Neutral*	0.074	0.051	0.098	< 0.001
Leaning Biden, *Discordant*	0.044	0.020	0.068	< 0.001
Leaning Trump, *Concordant*	0.023	–0.002	0.047	0.079
Leaning Trump, *Neutral*	0.042	0.016	0.068	0.004
Leaning Trump, *Discordant*	0.041	0.015	0.071	0.005
Community Flag [*ref.: Expert Flag*]	–0.013	–0.024	–0.002	0.017
Leaning Biden, *Concordant*	–0.010	–0.035	0.015	0.426
Leaning Biden, *Neutral*	–0.010	–0.032	0.015	0.404
Leaning Biden, *Discordant*	–0.010	–0.035	0.016	0.454
Leaning Trump, *Concordant*	–0.027	–0.052	–0.003	0.032
Leaning Trump, *Neutral*	–0.008	–0.034	0.022	0.504
Leaning Trump, *Discordant*	–0.013	–0.042	0.014	0.365
Leaning Trump [*ref.: Leaning Biden*]	–0.084	–0.092	–0.076	0.001
Concordant [*ref.: Neutral*]	–0.023	–0.032	–0.013	0.001
Discordant [*ref.: Neutral*]	0.022	0.011	0.032	< 0.001

Table S21. Average marginal effects (AME) and 95 % confidence intervals from a linear regression model with robust standard errors clustered on subjects and posts predicting the perceived misleadingness of a post (7-point Likert scale normalized to the interval [0, 1]). AME is the difference in average predicted probability of whether a fact-Check was rated as misleading between the group of interest and the reference group expressed as a proportion (e.g., an AME of $+0.05$ indicates a 5 percentage point difference in predicted probabilities). AMEs are reported separately for misleading (columns 2–5) and non-misleading posts (columns 6–9). The indented rows report the AMEs of an intervention depending on the political leanings of participants (leaning Trump vs. Biden) and the political congruence of the fact-Checked posts (concordant, neutral, discordant). Random intercepts for posts and subjects are included. The 95 % confidence intervals for the AMEs were derived using the bootstrap method for 1,000 resamples. $N =$ 64,454 observations across 1,810 participants.

	Misleading				Non-misleading			
	**AME**	**Lower CI**	**Upper CI**	**P-value**	**AME**	**Lower CI**	**Upper CI**	**P-value**
Expert Flag [*ref.: No Fact-Check*]	0.070	0.063	0.078	< 0.001	–0.052	–0.060	–0.044	0.001
Community Flag [*ref.: No Fact-Check*]	0.060	0.052	0.068	< 0.001	–0.051	–0.059	–0.042	0.001
Community Note [*ref.: No Fact-Check*]	0.096	0.088	0.104	< 0.001	–0.031	–0.039	–0.024	0.001
Community Note [*ref.: Expert Flag*]	0.026	0.018	0.033	< 0.001	0.020	0.013	0.028	< 0.001
Leaning Biden, *Concordant*	0.026	0.006	0.046	0.016	0.014	–0.004	0.031	0.123
Leaning Biden, *Neutral*	0.045	0.030	0.061	< 0.001	0.017	0.001	0.033	0.037
Leaning Biden, *Discordant*	0.005	–0.007	0.017	0.408	0.027	0.009	0.045	0.007
Leaning Trump, *Concordant*	0.044	0.022	0.065	< 0.001	0.035	0.014	0.055	< 0.001
Leaning Trump, *Neutral*	0.044	0.025	0.064	< 0.001	0.019	0.000	0.037	0.042
Leaning Trump, *Discordant*	–0.011	–0.028	0.006	0.188	0.009	–0.011	0.031	0.409
Community Flag [*ref.: Expert Flag*]	–0.010	–0.017	–0.003	0.010	0.001	–0.007	0.009	0.721
Leaning Biden, *Concordant*	–0.041	–0.064	–0.019	0.001	0.008	–0.011	0.026	0.386
Leaning Biden, *Neutral*	–0.016	–0.033	–0.001	0.036	–0.003	–0.018	0.014	0.743
Leaning Biden, *Discordant*	–0.002	–0.014	0.010	0.742	0.006	–0.014	0.026	0.534
Leaning Trump, *Concordant*	0.007	–0.015	0.029	0.501	–0.001	–0.022	0.020	0.945
Leaning Trump, *Neutral*	–0.004	–0.024	0.018	0.685	–0.009	–0.029	0.010	0.312
Leaning Trump, *Discordant*	–0.001	–0.017	0.015	0.911	0.007	–0.015	0.029	0.539
Leaning Trump [*ref.: Leaning Biden*]	–0.016	–0.022	–0.011	0.001	0.034	0.028	0.040	< 0.001
Concordant [*ref.: Neutral*]	–0.058	–0.065	–0.050	0.001	0.042	0.035	0.048	< 0.001
Discordant [*ref.: Neutral*]	0.092	0.086	0.098	< 0.001	0.168	0.161	0.175	< 0.001

C.7. Additional experiment for effects of presentation format. We conducted an additional experiment (see Section Effect of presentation format and context in the main paper) in which participants were randomly assigned to one of two conditions: (i) *community note (main)*, where misleading posts were supplemented with a textual community note without an explicit warning label (i.e., previous condition 4), or (ii) *community note (alternative)*, where misleading posts were supplemented by a combination of a textual community note and an explicit warning label indicating that community fact-checkers categorized the content as being misleading

We fitted hierarchical linear regression models to quantify the effects of the alternative presentation format of community notes on trust in fact-checks, and the identification of misleading and non-misleading posts. Note that due to the time that has passed since the first experiment was carried out and the resulting potential differences in the participants' level of knowledge on some of the topics covered in the social media posts, the answers cannot be directly compared across experiments. Therefore, we run a separate regression model for the new experiment. The key explanatory variable in the regression model was a binary dummy indicating the alternative presentation format. All other explanatory variables and random effects specifications were the same as in our main analysis. Tables S22 to S24 present the AMEs for our three dependent variables. The findings are described in Section Effect of presentation format and context of the main paper.

Table S22. Average marginal effects (AME) and 95 % confidence intervals from a hierarchical linear regression model with three-way interaction terms predicting the trustworthiness of a fact-check (7-point Likert scale normalized to the interval [0, 1]). AME is the difference in the average predicted trustworthiness ratings between the group of interest and the reference group (e.g., an AME of $+0.05$ indicates a 5 percentage point difference in trustworthiness ratings). The indented rows report the AMEs of an intervention depending on the political leanings of participants (leaning Trump vs. Biden) and the political congruence of the fact-checked posts (concordant, neutral, discordant). Random intercepts for posts and subjects are included. The 95 % confidence intervals for the AMEs were derived using the bootstrap method for 1,000 resamples. N = 12,150 observations across 675 participants.

	AME	Lower CI	Upper CI	P-value
Community Note (Alternative) [*ref.: (Main)*]	–0.001	–0.008	0.007	0.846
Leaning Biden	–0.026	–0.036	–0.016	0.001
Leaning Biden, *Concordant*	–0.024	–0.041	–0.004	0.017
Leaning Biden, *Neutral*	–0.030	–0.049	–0.013	0.001
Leaning Biden, *Discordant*	–0.024	–0.041	–0.008	0.005
Leaning Trump	0.025	0.015	0.036	< 0.001
Leaning Trump, *Concordant*	0.031	0.013	0.049	< 0.001
Leaning Trump, *Neutral*	0.017	–0.003	0.034	0.093
Leaning Trump, *Discordant*	0.029	0.009	0.048	< 0.001
Leaning Trump [*ref.: Leaning Biden*]	–0.063	–0.070	–0.055	0.001
Concordant [*ref.: Neutral*]	–0.026	–0.035	–0.017	0.001
Leaning Biden	–0.039	–0.053	–0.027	0.001
Leaning Trump	–0.012	–0.026	0.001	0.067
Discordant [*ref.: Neutral*]	0.011	0.002	0.020	0.010
Leaning Biden	0.020	0.009	0.032	< 0.001
Leaning Trump	0.001	–0.012	0.014	0.858

Table S23. Average marginal effects (AME) and 95 % confidence intervals from a hierarchical linear regression model with four-way interaction terms predicting the perceived misleadingness of a post (7-point Likert scale normalized to the interval [0, 1]). AME is the difference in the average predicted misleadingness ratings between the group of interest and the reference group (e.g., an AME of $+0.05$ indicates a 5 percentage point difference in misleadingness ratings). AMEs are reported separately for misleading (columns 2–5) and non-misleading posts (columns 6–9). The indented rows report the AMEs of an intervention depending on the political leanings of participants (leaning Trump vs. Biden) and the political congruence of the fact-checked posts (concordant, neutral, discordant). Random intercepts for posts and subjects are included. The 95 % confidence intervals for the AMEs were derived using the bootstrap method for 1,000 resamples. N = 24,300 observations across 675 participants.

	**Misleading**				**Non-misleading**			
	**AME**	**Lower CI**	**Upper CI**	**P-value**	**AME**	**Lower CI**	**Upper CI**	**P-value**
Community Note (Alternative) [*ref.: (Main)*]	0.020	0.012	0.027	< 0.001	–0.014	–0.023	–0.005	0.001
Leaning Biden	0.014	0.004	0.024	0.011	–0.012	–0.023	0.000	0.050
Leaning Biden, *Concordant*	0.010	–0.008	0.028	0.292	–0.002	–0.023	0.018	0.828
Leaning Biden, *Neutral*	0.008	–0.010	0.025	0.368	–0.008	–0.027	0.010	0.399
Leaning Biden, *Discordant*	0.023	0.006	0.039	0.005	–0.023	–0.045	–0.002	0.032
Leaning Trump	0.026	0.015	0.037	< 0.001	–0.017	–0.028	–0.004	0.011
Leaning Trump, *Concordant*	0.046	0.026	0.068	< 0.001	–0.018	–0.039	0.003	0.113
Leaning Trump, *Neutral*	0.014	–0.005	0.033	0.122	0.002	–0.020	0.023	0.865
Leaning Trump, *Discordant*	0.017	0.000	0.034	0.051	–0.030	–0.052	–0.008	0.011
Leaning Trump [*ref.: Leaning Biden*]	–0.010	–0.017	–0.002	0.016	0.019	0.010	0.027	< 0.001
Concordant [*ref.: Neutral*]	–0.048	–0.057	–0.038	0.001	0.051	0.041	0.061	< 0.001
Leaning Biden	–0.080	–0.092	–0.068	0.001	0.031	0.016	0.045	< 0.001
Leaning Trump	–0.015	–0.029	–0.001	0.030	0.072	0.056	0.087	< 0.001
Discordant [*ref.: Neutral*]	0.032	0.023	0.040	< 0.001	0.153	0.143	0.163	< 0.001
Leaning Biden	0.035	0.024	0.047	< 0.001	0.148	0.133	0.162	< 0.001
Leaning Trump	0.029	0.016	0.042	< 0.001	0.158	0.144	0.173	< 0.001

Table S24. Average marginal effects (AME) and 95 % confidence intervals from a hierarchical linear regression model with four-way interaction terms predicting sharing intentions (7-point Likert scale normalized to the interval [0, 1]). AME is the difference in average predicted sharing intentions between the group of interest and the reference group (e.g., an AME of $+0.05$ indicates a 5 percentage point difference in sharing intentions). AMEs are reported separately for misleading (columns 2–5) and non-misleading posts (columns 6–9). The indented rows report the AMEs of an intervention depending on the political leanings of participants (leaning Trump vs. Biden) and the political congruence of the fact-checked posts (concordant, neutral, discordant). Random intercepts for posts and subjects are included. The 95 % confidence intervals for the AMEs were derived using the bootstrap method for 1,000 resamples. $N=$ 24,300 observations across 675 participants.

	Misleading				Non-misleading			
	**AME**	**Lower CI**	**Upper CI**	**P-value**	**AME**	**Lower CI**	**Upper CI**	**P-value**
Community Note (Alternative) [*ref.: (Main)*]	–0.009	–0.016	–0.002	0.005	0.012	0.004	0.020	0.006
Leaning Biden	–0.021	–0.031	–0.012	0.001	0.015	0.005	0.026	0.006
Leaning Biden, *Concordant*	–0.015	–0.031	0.001	0.062	0.003	–0.017	0.024	0.764
Leaning Biden, *Neutral*	–0.024	–0.040	–0.008	0.006	0.022	0.003	0.042	0.024
Leaning Biden, *Discordant*	–0.023	–0.038	–0.008	0.003	0.019	0.001	0.037	0.024
Leaning Trump	0.003	–0.006	0.013	0.521	0.009	–0.002	0.020	0.150
Leaning Trump, *Concordant*	–0.001	–0.017	0.015	0.872	–0.001	–0.021	0.020	0.955
Leaning Trump, *Neutral*	0.014	–0.004	0.031	0.112	–0.002	–0.024	0.018	0.873
Leaning Trump, *Discordant*	–0.003	–0.020	0.013	0.706	0.029	0.010	0.048	< 0.001
Leaning Trump [*ref.: Leaning Biden*]	0.014	0.007	0.021	< 0.001	–0.002	–0.010	0.006	0.579
Concordant [*ref.: Neutral*]	0.010	0.002	0.019	0.022	–0.003	–0.014	0.008	0.583
Leaning Biden	0.009	–0.002	0.021	0.114	0.009	–0.005	0.024	0.241
Leaning Trump	0.010	–0.002	0.022	0.096	–0.015	–0.030	0.000	0.049
Discordant [*ref.: Neutral*]	–0.006	–0.014	0.002	0.150	–0.072	–0.081	–0.062	0.001
Leaning Biden	–0.009	–0.021	0.003	0.150	–0.076	–0.090	–0.063	0.001
Leaning Trump	–0.003	–0.015	0.008	0.599	–0.067	–0.081	–0.054	0.001

D. Further methodological details

D.1. Participants. Our preregistration (which can be found here: https://aspredicted.org/rb45k.pdf) describes that we "plan to recruit 1500 participants." In the end, we slightly exceeded this number: after handling all outliers 1810 valid participants remained (1,347 in treatment conditions). Participants were recruited between April 30th and June 15th in seven experimental sessions to achieve a balanced number of participants with different political leanings (Republicans are underrepresented on Prolific (8)). Specifically, we explicitly recruited participants who indicated they voted for Biden (sessions 2, 5, and 6) or Trump (sessions 3, 4, and 7) in the 2020 presidential election using the prescreening tool available on Prolific. Participants who participated in one session were prevented from participating in subsequent session. The procedure was identical in all sessions. In our regression analysis, we control for potential pre-treatment effects by including random intercepts for subjects and posts. Participants were paid $4 for completing the survey (approx. 20min), which translated to an hourly wage of $12/h.

The participants were allocated to the individual sessions as follows:

- Session 1: April 30th, 2023. $n = 296$ finished the survey, participants who indicated responding randomly ($n = 8$), searching online for any of the headlines during the experiment ($n = 6$), do not have a social media account ($n = 9$), or failed the attention checks ($n = 29$) were removed from analysis. The final sample was $n = 260$ ($M_{Age} = 37$, 52 % female).

- Session 2: May 15th, 2023. $n = 397$ finished the survey, participants who indicated responding randomly ($n = 5$), searching online for any of the headlines during the experiment ($n = 6$), do not have a social media account ($n = 5$), or failed the attention checks ($n = 19$) were removed from analysis. The final sample was $n = 374$ ($M_{Age} = 41$, 51 % female).

- Session 3: May 16th, 2023. $n = 403$ finished the survey, participants who indicated responding randomly ($n = 5$), searching online for any of the headlines during the experiment ($n = 4$), do not have a social media account ($n = 11$), or failed the attention checks ($n = 30$) were removed from analysis. The final sample was $n = 362$ ($M_{Age} = 46$, 52 % female).

- Session 4: May 25th, 2023. $n = 260$ finished the survey, participants who indicated responding randomly ($n = 4$), searching online for any of the headlines during the experiment ($n = 1$), do not have a social media account ($n = 7$), or failed the attention checks ($n = 25$) were removed from analysis. The final sample was $n = 227$ ($M_{Age} = 43$, 53 % female).

- Session 5: May 26th, 2023. $n = 156$ finished the survey, participants who indicated responding randomly ($n = 2$), searching online for any of the headlines during the experiment ($n = 2$), do not have a social media account ($n = 6$), or failed the attention checks ($n = 13$) were removed from analysis. The final sample was $n = 137$ ($M_{Age} = 40$, 48 % female).

- Session 6: June 14th, 2023. $n = 252$ finished the survey, participants who indicated responding randomly ($n = 1$), searching online for any of the headlines during the experiment ($n = 3$), do not have a social media account ($n = 5$), or failed the attention checks ($n = 18$) were removed from analysis. The final sample was $n = 230$ ($M_{Age} = 40$, 49 % female).

- Session 7: June 15th, 2023. $n = 250$ finished the survey, participants who indicated responding randomly ($n = 2$), searching online for any of the headlines during the experiment ($n = 4$), do not have a social media account ($n = 10$), or failed the

attention checks ($n = 21$) were removed from analysis. The final sample was $n = 220$ ($M_{Age} = 45$, 51 % female).

D.2. Participants in additional experiment. For the additional experiment studying the effects of different presentation formats of community notes, we recruited $n = 795$ participants between February 6th and February 13th, 2024 in one experimental session via Prolific.com. Survey design, prescreening, payment, and data cleansing were conducted identically to the main experiment. In addition, we excluded people who had already taken part in the survey last year.

Participants who indicated responding randomly ($n = 19$), searching online for any of the headlines during the experiment ($n = 26$), not having a social media account ($n = 15$), or failed the attention checks ($n = 64$) were removed from analysis. This led to a final sample of $n = 675$ participants ($M_{Age} = 42.88$, 50.81 % female).

D.3. Additional question items. Following the questions regarding trustworthiness, misleadingness, and sharing intentions, participants were asked to complete a 4-item Cognitive Reflection Test (CRT). A CRT is a common method to determine a person's level of reflective thinking. The purpose of its design is to assess an individual's inclination to replace an intuitive yet incorrect response with a more rational and accurate response (9). For our CRT, we used a combination of both numeric and non-numeric questions from different sources (9, 10) (see Figure S6).

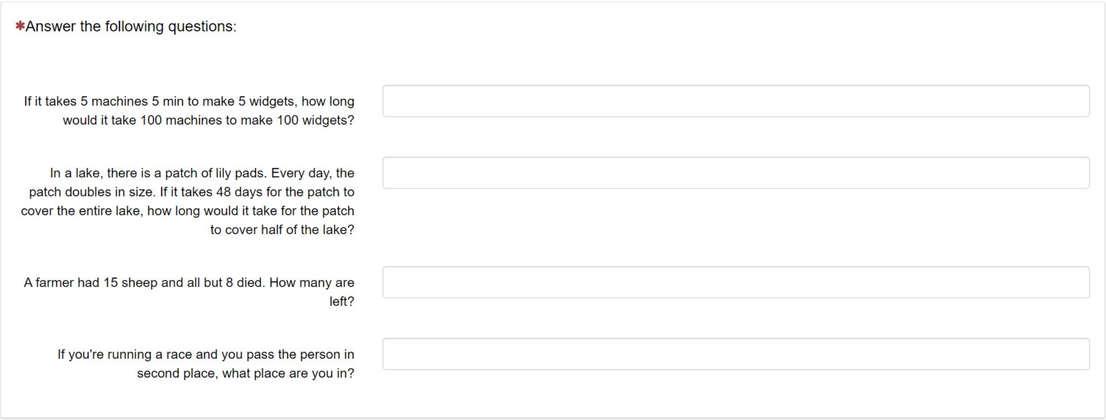

Fig. S6. CRT Questions

Subsequently, participants were asked about their social media use:

- What type of social media accounts do you use (if any)? (Facebook, Twitter, Snapchat, Instagram, WhatsApp, TikTok, Other, None)
- Which of these types of content would you consider sharing on social media (if any)? (Political news, Sports news, Celebrity news, Science/technology news, Business news, Other, None)
- When deciding whether to share a piece of content on social media, how important is it to you that the content is... (Accurate, Surprising, Interesting, Aligned with my beliefs, Funny; each answered on a 5-point likert scale (Not al all, Slightly, Moderately, Very, Extremely))

- To what extend do you trust the information that comes from the following? (National news organizations, local news organization, friends and family, Social network sites (e.g., Facebook, Twitter), 3rd party fact-checkers (e.g., snopes.com, factcheck.org); each answered on a 5-point likert scale (not at all, a little, a moderate amount, a lot, a great deal))

Depending on the condition participants where assigned to (condition 2–4), we also asked questions about their awareness of the specific type of fact-checks and what influence the respective fact-checks had on their assessment regarding the accuracy of a post:

- Prior to you taking this study, were you aware of the existence of third-party fact-checking organizations (community-based fact-checking)? (Yes/No)

- To what extent did the “Checked by third-party fact-checking organizations” tag (“Checked by other social media users with multiple perspectives tag”, “Community note”) influence you opinion about the accuracy of the social media posts? (5-point likert scale; Not at all, Slightly, Moderately, Very, Extremely)

- We are interested in whether the “Checked by third-party fact-checking organizations” tag (“Checked by other social media users with multiple perspectives tag”, “Community note”) influenced your opinion about the accuracy of the social media posts that were tagged as potentially misleading. I rated "potentially misleading" posts as: (7-point likert scale; Much less accurate, Less accurate, Slightly less accurate, Tag had no influence, Slightly more accurate, More accurate, Much more accurate)

- We are interested in whether the “Checked by third-party fact-checking organizations” tag (“Checked by other social media users with multiple perspectives tag”, “Community note”) influenced your opinion about the accuracy of the social media posts that were NOT tagged as potentially misleading. I rated posts that were NOT "potentially misleading" as: (7-point likert scale; Much less accurate, Less accurate, Slightly less accurate, Tag had no influence, Slightly more accurate, More accurate, Much more accurate)

At the end of the survey, participants where asked to answer several demographic questions: age, gender, level of education, proficiency in English, US region where they live, stance toward god (or gods), whether they have been vaccinated against COVID-19, whether they see themselves as part of an ethnic minority, political orientation (Democrat, Republican, Third Party, Other), and questions on their voting behavior in the 2020 presidency election. First, they were asked who they voted for (Joe Biden, Donald Trump, Other Candidate, I did not vote for reasons outside my control, I did not vote but I could have, I did not vote out of protest) and second, who they would prefer to be president, if they absolutely had to choose between Joe Biden and Donald Trump.

In addition, participants had to provide an assessment of their attitudes toward the following statements on risk aversion and trust (5-point likert scale; Not at all, Not really, Undecided, Somewhat, Very much):

- I am generally a person that is fully prepared to take risks.

- I usually have the feeling that I can trust the people I interact with in my daily life.

- I have a fundamental trust in democracy.

- I would rather do something that requires little thought than something that is sure to challenge my thinking abilities.

At the end of the survey, participants were asked if they responded randomly at any point during the survey or searched for the content online. Participants who answered “yes” to any of these questions were excluded from the analysis. Furthermore, we excluded participants who indicated not to have any social media account or failed any attention check.

E. Selection of social media posts and fact-checks

All participants were presented with 36 social media posts (18 "Non-misleading" and 18 "misleading"). The posts for our study were selected as follows. First, misleading posts and corresponding fact-checks have been manually selected from X's Community Notes platform. Only posts that had been identified as helpful by the platform's bridging algorithm (11) – meaning they were rated as helpful by users across diverse viewpoints and made visible on X (https://communitynotes.x.com/guide/en/contributing/notes-on-twitter) – were considered. Two research assistants (RAs) initially selected around 50 posts to cover a diverse set of posts across a wide variety of relevant topics (Politics, Business, Health, Climate Change, Celebrities, Other). From this initial selection, 18 posts were chosen for our study to appeal to subjects with different political views (6 "pro-Democrat," 6 "Neutral," and 6 "pro-Republican" posts). We aimed for a balanced distribution of the political leanings within the individual topic groups, where appropriate. For example, the topics "Politics" and "Health" comprised posts across all political leanings, whereas posts on "Celebrities" were all politically neutral. Second, Non-misleading posts have been manually selected via corresponding keyword searches from X to represent a similar distribution of topics. Here, we again started with a larger list of posts, which was then narrowed down to 18 posts that were evenly distributed across political lines (6 "pro-Democrat," 6 "Neutral," and 6 "pro-Republican" posts).

To ensure the correctness of the fact-checking labels, we have taken the following steps. First, all fact-checks for misleading posts were carried out by community fact-checkers on X's Community Notes platform and rated as helpful. Second, we ensured that we only include posts that we perceived as clearly misleading or non-misleading. Third, we had three trained RAs manually assess the veracity of the posts. The RAs were not aware of the fact-checking label and were tasked to manually assess professional fact-checks (snopes.com, factcheck.org, etc.) or other reliable sources to determine whether the posts were misleading (or Non-misleading). The assessments of the RAs were in perfect agreement with the fact-checking labels.

As a further check, we had three trained RA to validate our labels for the political orientation of the posts. The RAs were asked to assume that the content of all posts was entirely accurate and to evaluate whether the posts would have been more favorable to Democrats or Republicans (on a 5-point Likert-scale ranging from "more favorable to Democrats" (1) to "more favorable to Republicans" (5)). The distribution of participant's responses is shown in Figure S7. Overall, pro-Democrat posts ($M_{Misleading/Dem} = 1.40$, $M_{Non-misleading/Dem} = 1.53$) were rated as significantly less favorable for Republicans than pro-Republican posts ($M_{Misleading/Rep} = 4.47$, $M_{Non-misleading/Rep} = 4.10$). Pro-Democrat posts were rated as significantly more favorable to Democrats than politically neutral posts and pro-Republican posts were more favorable to Republicans than politically neutral posts. Statistically, each of these differences in means was significant according to two-sided t-tests (each $P < 0.001$).

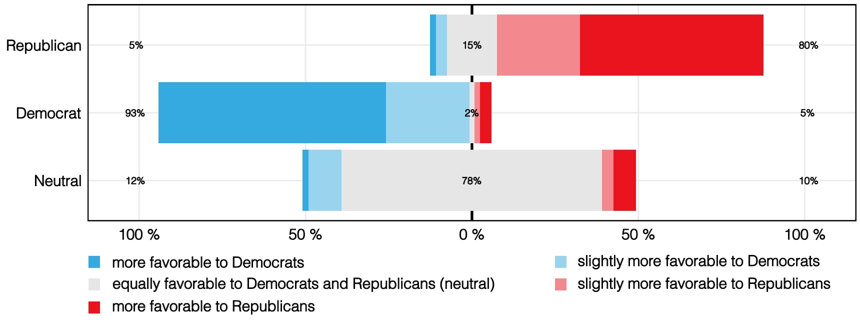

Fig. S7. Distribution of participants' responses regarding the political orientation of posts on 5-point Likert scales.

References

1. John Gramlich. What the 2020 electorate looks like by party, race and ethnicity, age, eduction and religion. https://www.pewresearch.org/short-reads/2020/10/26/what-the-2020-electorate-looks-like-by-party-race-and-ethnicity-age-education-and-religion/, 2020.
2. Carroll Doherty, Jocelyn Kiley, and Olivia O'Hea. Wide gender gap, growing educational divide in voters' party identification. https://www.pewresearch.org/politics/2018/03/20/1-trends-in-party-affiliation-among-demographic-groups/, 2018.
3. Xinyuan Ye. Exploring the relationship between political partisanship and COVID-19 vaccination rate. *Journal of Public Health*, 45(1):91–98, 10 2021. ISSN 1741-3842. . URL https://doi.org/10.1093/pubmed/fdab364.
4. Giancarlo Pasquini and Emily Saks. Partican differences are common in the lessons americans take away from Covid-19. https://www.pewresearch.org/short-reads/2022/09/06/partisan-differences-are-common-in-the-lessons-americans-take-away-from-covid-19/, 2022.
5. Poynter. Most Republicans don't trust fact-checkers, and most Americans don't trust the media. https://www.poynter.org/ifcn/2019/most-republicans-dont-trust-fact-checkers-and-most-americans-dont-trust-the-media/, 2019.
6. Ziv Epstein, Nathaniel Sirlin, Antonio Arechar, Gordon Pennycook, and David Rand. The social media context interferes with truth discernment. *Science Advances*, 9(9):eabo6169, 2023.
7. Gordon Pennycook, Adam Bear, Evan T. Collins, and David G. Rand. The implied truth effect: Attaching warnings to a subset of fake news headlines increases perceived accuracy of headlines without warnings. *Management Science*, 66(11):4944–4957, 2020.
8. Benjamin D. Douglas, Patrick J. Ewell, and Markus Brauer. Data quality in online human-subjects research: Comparison between MTurk, Prolific, CloudResearch, Qualtrics, and SONA. *PLOS ONE*, 18(3):e0279720, 2023. .
9. Shane Frederick. Cognitive reflection and decision making. *Journal of Economic Perspectives*, 19(4):25–42, 2005.
10. Keela S Thomson and Daniel M Oppenheimer. Investigating an alternate form of the cognitive reflection test. *Judgment and Decision Making*, 11:99–113, 2016.
11. Stefan Wojcik, Sophie Hilgard, Nick Judd, Delia Mocanu, Stephen Ragain, MB Hunzaker, Keith Coleman, and Jay Baxter. Birdwatch: Crowd wisdom and bridging algorithms can inform understanding and reduce the spread of misinformation. *arXiv*, 2022.
